# Phenotypic and functional characterization of T cells in white matter lesions of multiple sclerosis patients

**DOI:** 10.1007/s00401-017-1744-4

**Published:** 2017-06-17

**Authors:** Gijsbert P. van Nierop, Marvin M. van Luijn, Samira S. Michels, Marie-Jose Melief, Malou Janssen, Anton W. Langerak, Werner J. D. Ouwendijk, Rogier Q. Hintzen, Georges M. G. M. Verjans

**Affiliations:** 1 Department of Viroscience, Erasmus MC, University Medical Center, Room Ee1720a, ‘s-Gravendijkwal 230, 3015 CE Rotterdam, The Netherlands; 2 Department of Neurology, Erasmus MC, University Medical Center, Rotterdam, The Netherlands; 3 Department of Immunology, Erasmus MC, University Medical Center, Rotterdam, The Netherlands; 4 MS Center ErasMS, Erasmus MC, University Medical Center, Rotterdam, The Netherlands; 5 Research Center for Emerging Infections and Zoonoses, University of Veterinary Medicine, Hannover, Germany

**Keywords:** Multiple sclerosis, Pathogenesis, CD8 T cells, Autoantigens, Epstein–Barr virus

## Abstract

T cells are considered pivotal in the pathology of multiple sclerosis (MS), but their function and antigen specificity are unknown. To unravel the role of T cells in MS pathology, we performed a comprehensive analysis on T cells recovered from paired blood, cerebrospinal fluid (CSF), normal-appearing white matter (NAWM) and white matter lesions (WML) from 27 MS patients with advanced disease shortly after death. The differentiation status of T cells in these compartments was determined by ex vivo flow cytometry and immunohistochemistry. T-cell reactivity in short-term T-cell lines (TCL), generated by non-specific stimulation of T cells recovered from the same compartments, was determined by intracellular cytokine flow cytometry. Central memory T cells predominated in CSF and effector memory T cells were enriched in NAWM and WML. WML-derived CD8^+^ T cells represent chronically activated T cells expressing a cytotoxic effector phenotype (CD95L and granzyme B) indicative for local antigenic stimulation (CD137). The same lesions also contained higher CD8^+^ T-cell frequencies expressing co-inhibitory (TIM3 and PD1) and co-stimulatory (ICOS) T-cell receptors, yet no evidence for T-cell senescence (CD57) was observed. The oligoclonal T-cell receptor (TCR) repertoire, particularly among CD8^+^ T cells, correlated between TCL generated from anatomically separated WML of the same MS patient, but not between paired NAWM and WML. Whereas no substantial T-cell reactivity was detected towards seven candidate human MS-associated autoantigens (cMSAg), brisk CD8^+^ T-cell reactivity was detected in multiple WML-derived TCL towards autologous Epstein–Barr virus (EBV) infected B cells (autoBLCL). In one MS patient, the T-cell response towards autoBLCL in paired intra-lesional TCL was dominated by TCRVβ2^+^CD8^+^ T cells, which were localized in the parenchyma of the respective tissues expressing a polarized TCR and CD8 expression suggesting immunological synapse formation in situ. Collectively, the data suggest the involvement of effector memory cytotoxic T cells recognizing antigens expressed by autoBLCL, but not the assayed human cMSAg, in WML of MS patients.

**Electronic supplementary material:**

The online version of this article (doi:10.1007/s00401-017-1744-4) contains supplementary material, which is available to authorized users.

## Introduction

Multiple sclerosis (MS) is a chronic debilitating disease characterized by inflammation of the central nervous system (CNS), leading to demyelination and axon damage. The cause and pathogenic pathways involved remain enigmatic. MS is considered the result of a local inflammatory response in genetically susceptible individuals initiated by environmental factors [12, 17].

MS immunopathology has been widely studied in experimental autoimmune encephalomyelitis (EAE) animal models. EAE is induced by immunization with candidate MS-associated CNS autoantigens (cMSAg) in adjuvant and by adoptive transfer of cMSAg-specific CD4^+^ T cells. These studies reinforced the idea that MS is an autoimmune disease mediated by cMSAg-specific CD4^+^ Th1/Th17 cells [28]. Indeed, CD4^+^ T-cell responses to the majority of EAE-inducing cMSAg have been identified in peripheral blood (PB) and occasionally CSF of MS patients [17, 18, 28]. However, their frequency in PB of MS patients was indifferent compared to controls, questioning their pathogenic potential [17, 19]. The additional role of CD8^+^ T cells in MS and EAE immunopathology has only recently emerged [19, 28]. In MS patients, CD8^+^ T cells outnumber CD4^+^ T cells in active lesions and closely interact with oligodendrocytes and demyelinating axons [12, 19, 41]. CD8^+^ T cells infiltrates in active white matter lesions (WML) are oligoclonal and persist in CSF and peripheral blood [5, 48, 55]. Human cMSAg-specific CD8^+^ T cells lyse human leukocyte antigen (HLA) class I-matched oligodendrocytes in vitro [26, 35, 57]. The cytotoxic effector mechanisms involve both the Fas/FasL and perforin/granzyme B (grB) pathways [11, 40, 50, 57].

Current data advocate Epstein–Barr virus (EBV) infection as risk factor in MS [33, 39]. The epidemiological similarity of MS and EBV-induced infectious mononucleosis (IM), enhanced systemic B- and T-cell responses to EBV in MS patients compared to (disease) controls, and the putative presence of EBV-infected B cells in MS lesions are indicative [4, 43, 53]. Even though evidence for the presence of EBV in MS lesions remains incredulous, [45] EBV may evoke T-cell-mediated MS immunopathology in different ways [8, 20]. Virus-specific T cells may recognize EBV-infected cells in the brain or alternatively cross-react with cMSAg [61]. Alternatively, IM-associated systemic immune activation may result in a cytokine environment that activates pathogenic T cells, including cMSAg-specific T cells, in an antigen-independent manner [33, 39].

The aims of this study were to characterize the differentiation status and antigen specificity of T cells recovered from paired white matter tissues containing WML, normal-appearing white matter (NAWM), CSF and PB from 27 patients with advanced MS.

## Materials and methods

### Clinical specimens

Heparinized PB (3–6 ml; *n* = 19), CSF (8–20 ml; *n* = 17), and macroscopically defined NAWM (*n* = 23) and WML (*n* = 29), were obtained from 27 MS patients (median age 59 years, range 35–95 years) at autopsy with a median post-mortem interval of 9.6 h (range 4.8–14 h) (Online Resource 1). All patients had advanced disease (median disease duration 25 years, range 10–54 years), expanded disability status scale >6 and the majority had primary or secondary progressive MS (22 of 25, 88%). Leading cause of death was legally granted euthanasia for 11 of 27 (44%) patients. Additionally, 12 formalin-fixed and paraffin-embedded (FFPE) tissues of 10 diseased MS patients were collected (Online Resource 2). Clinical specimens of deceased MS patients were collected by the Netherlands Brain Bank (NBB; Amsterdam, The Netherlands) and genital skin biopsies of six patients with herpes simplex virus type 2 (HSV-2) genital herpes (GH) were obtained by the department of Dermatology and Venereology (Erasmus MC, Rotterdam, The Netherlands). Written informed consent for brain autopsy and genital skin biopsies, use of clinical specimens and clinical information for research purposes have been obtained in advance from all study participants. Study procedures were performed in compliance with Dutch legislation and institutional guidelines, approved by the respective local ethical committees for use of MS patient material (Project Number 2009/148; VU University Medical Center, Amsterdam) and skin biopsies of GH patients (Project Number MEC 167.153/1998/15; Erasmus MC) performed in accordance with the ethical standards as laid down in the 1964 Declaration of Helsinki and its later amendments or comparable ethical standards. During autopsy, WML was distinguished macroscopically from NAWM based on gray appearance and by being firm to touch. PB was used to generate EBV-transformed B-cell lines (BLCL) and for 2-digit HLA typing as described [60]. Part of white matter tissues (1–4 g) were snap-frozen and stored at −80 °C for subsequent in situ analysis. Remaining tissue was dispersed to single cell suspensions as described [60]. About one-tenth of the single cell suspension was snap-frozen for subsequent EBV-specific EBER1 transcript expression analysis and remaining cells were used for ex vivo flow cytometric analysis and to generate short-term T-cell lines (TCL) as described [60]. In brief, CSF and lymphocyte-enriched brain tissue cell suspensions were stimulated with phytohemagglutinin-L (1 µg/ml; Roche, Branford, CT) for 10–14 days and subsequently with an anti-CD3 monoclonal antibody (mAb) (clone OKT-3; Janssen-Cilag, Tilburg, The Netherlands) for 10–14 days in the presence of γ-irradiated (3000 rad) allogeneic PBMC and recombinant human interleukin 2 (rIL-2; 50 IU/ml) and rIL-15 (25 ng/ml; both Miltenyi Biotec, Bergisch Gladbach, Germany) [43].

### In situ analyses of brain tissues

To classify the WM tissues of MS patients, brain tissues were characterized by immunohistochemistry (IHC) as NAWM, diffuse white matter abnormalities (DWMA), active lesions (AL), mixed active/inactive lesions (mAIL) and inactive lesions (IL) as described in the recently updated classification system for MS brain lesions [29] (representative stainings of defined WM tissues of MS patients are shown in Online Resource 3). In brief, consecutive 7- to 9-µm cryostat brain sections were air-dried, fixed with acetone and subsequently assayed for: (1) (de-)myelination by anti-myelin oligodendrocyte protein (MOG) (clone Z12; kindly provided by prof. Sandra Amor, VU Medical Center, Amsterdam); (2) immune activation using anti-HLA-DP/DQ/DR mAb (CR3/43; Dako, Glostrup, Denmark); (3) presence of macrophages and microglia using anti-CD68 (clone EMB11; Dako). Granzyme B expressing T cells were detected in consecutive 6-µm sections of FFPE mAIL tissue of four representative MS patients stained for CD3 (clone F7.2.38), CD8 (C8/144B), grB (GrB-7, all Dako). Stainings were visualized by 3-amino-9-ethylcarbazole as described [60]. Intracellular localization of grB and cytotoxic potential of CD8^+^ T cells was assessed in 8-µm FFPE sections by triple immunofluorescent stainings using mAb directed to CD8 (clone YTC182.20, AbD Serotec), grB and cleaved caspase 3 (cCASP3; clone G7481, Promega, Madison, USA). The presence of tissue-resident T cells (T_RM_) was assessed analogously by detecting CD8, CD69 (FN50, Biolegend) and CD103 (2G5.1, Thermo Fisher) expression in WML of MS patients and as control skin biopsies of GH patients. CD8^+^ T cells expressing grB in the parenchyma and perivascular space of mAIL sections were counted in multiple z-stack scans acquired at 400× magnification. To determine the CNS cell types encountered by intra-lesional CD8^+^ T cells, double-immunofluorescence stainings were performed on 8-μm FFPE sections of WML biopsies. CNS cell types were visualized using mAbs directed to neuron-specific neurofilament heavy chain (NF-H, clone SMI-31, Biolegend), microglia-specific ionized calcium-binding adapter molecule 1 (Iba1; clone 019-19741, Wako, Osaka, Japan), oligodendrocyte-specific proteolipid protein (PLP; clone plpc1, AbD Serotec) and astrocyte-specific glial fibrillary acidic protein (GFAP; clone Z0334, Dako) combined with an anti-CD8 mAb (C8/144B). Antibody binding was visualized by AF488-conjugated Rat IgG (A-11006), AF568-conjugated IgG1 (A-21124), AF488- or AF647-conjugated IgG2a (A-21131 or A-21241) or AF647-conjugated rabbit IgG-specific (A-21246, all Invitrogen) antibodies. To detect T-cell receptor beta chain 2 (TCRVβ2) expressing T cells in situ, 8-µm cryostat brain sections were stained with APC-conjugated CD8 (RPA-T8, BD), FITC-conjugated TCRVβ2 (MPB2D5; Beckman Coulter) and laminin (L9393; Sigma) mAb combined with AF594-conjugated anti-rabbit IgG (R37119; Invitrogen). TCRVβ2 staining was amplified using the FASER kit (Miltenyi Biotec). For all fluorescent stainings, nuclei were stained with 4′,6-diamidino-2-phenylindole (DAPI) to visualize nuclei and sections scanned using LSM700 confocal microscope and Zen 2010 software (Zeiss; Oberkochen, Germany). Appropriate (fluorochrome-) matched Ig isotype antibodies were used as negative controls for all stainings and stainings scored by two independent observers.

### Ex vivo T-cell phenotyping

Paired PB-, CSF-, NAWM- and WML-derived cells were incubated with combinations of fluorochrome-conjugated mAb directed to following markers: CD3 (clone UCHT1), CD4 (SK3 or RPA-T4), CD8 (SK1 or RPA-T8), CD27 (M-T271), CD45 (TU116), CD56 (B159), CD137 (4-1BB; all Becton Dickinson (BD), Franklin Lakes, NJ), CD57 (HCD57), CD95L (NOK-1), ICOS (C398.4A), PD1 (EH12.2H7), TIM3 (F38-2E2; all Biolegends, San Diego, CA) and CD45RA (HI100; Pharmingen, San Diego, CA). Cells were measured on a Canto II flow cytometer (BD). Negative controls consisted of stainings with appropriate Ig isotype control antibodies. If a parent population contained <100 events, the gated cell population was omitted from further analysis.

### Quantitative EBV transcript expression analysis

Snap-frozen brain tissue-derived single cell pellets were used for RNA isolation and cDNA synthesis as described [60]. EBV EBER1 transcript expression was quantified by real-time quantitative PCR (qPCR) using forward primer 5′-TCATAGGGAGGAGACGTGTGT-3′, reverse primer 5′-TGACCGAAGACGGCAGAAAG-3′ and probe 5'FAM-AGACAACCACAGACACCGTGGTGACCA-3′MGB (all Eurogentec; Liege, Belgium). RNA isolated from water and BLCL served as negative and positive controls in each run, respectively. For each sample β actin transcripts (Hs01060665_g1, Applied Biosystems) were used as positive control for RNA isolation and cDNA synthesis. Sensitivity of the EBER1-specific qPCR was determined as 1–10 BLCL per 10^6^ PBMC (data not shown). An ABI prism 7500 and Taqman Universal Master Mix (both Applied Biosystems; Nieuwerkerk, The Netherlands) were used for all qPCR reactions as described [60].

### Functional T-cell assays

T-cell reactivity towards EBV-infected B cells in CSF-, NAWM- and WML-TCL was determined by incubation with autologous EBV-transformed B-cell lines (autoBLCL) as antigen presenting cell (APC) as described previously [43]. The frequency of B cells in BLCL lines (*n* = 5) undergoing spontaneous lytic cycle infection was determined by flow cytometric analysis of EBV glycoprotein 350 expression (clone OT.1C-2, kindly provided by prof. J.M. Middeldorp, VU Medical Center, Amsterdam, The Netherlands). An allogeneic BLCL (BLCL-GR; HLA-A*01;03, -B*07;27, -C*02;07, -DRB1*13;15, -DQB1*06;06), carrying the major MS-associated HLA class I and II risk alleles (HLA-A*03, -DRB1*15 and -DRB1*13) [49], was transduced to express the seven following human cMSAg constitutively: (1) oligodendrocyte-specific proteins [myelin-associated glycoprotein (MAG), myelin basic protein isoform 1 (MBP1) and myelin oligodendrocyte glycoprotein (MOG)]; (2) neuron-specific proteins [contactin-2 (CNTN2) and 155-kDa isoform of neurofascin (NFASC)] and (3) glia-specific proteins [inwards rectifying potassium channel (KIR4.1) and S100 calcium-binding protein B (S100B)] [42]. Stable cMSAg-expressing BLCL-GR lines, validated by flow cytometry using cMSAg-specific mAb, were used as allogeneic APC to detect cMSAg-specific CD4^+^ and CD8^+^ T cells simultaneously in TCL of HLA-matched MS patients (Online Resource 1) [42]. To validate this BLCL platform, we also transduced BLCL-GR to express measles virus fusion protein (MVF) and assayed their APC function by incubation with MVF-specific CD4^+^ (4-F99) and CD8^+^ (2-F40) T-cell clones (TCC) as described previously [43]. Stimulated T cells were stained with fluorochrome-conjugated mAb to human CD3 (SP34-2), CD4 (SK3), CD8α (RPA-T8) and for viability (violet live/dead stain; Invitrogen). Subsequently, cells were fixed, permeabilized and stained for intracellular IFNγ (B27) and CD137 (4-1BB, all BD) and finally measured on a Canto II flow cytometer. Combined intracellular IFNγ and CD137, two independent T-cell activation markers induced upon antigenic stimulation, was set as criterion to identify antigen-specific T cells [10, 43]. TCL stimulated with phorbol myristate-acetate (PMA; 50 ng/ml) and ionomycin (Iono; 500 ng/ml; both Sigma), or mock-stimulated TCL, were used as positive and negative control, respectively. Netto reactivity of CD4^+^ and CD8^+^ T cells towards cMSAg-transduced BLCL-GR was calculated by subtracting T-cell reactivity to cMSAg-transduced BLCL-GR with mock-transduced BLCL-GR. Threshold for positive cMSAg T-cell reactivity was calculated as previously reported [42]. Experiments were performed on at least two independent occasions.

### T-cell receptor repertoire analysis

The clonality of NAWM- and WML-derived TCL was determined using a set of fluorochrome-conjugated mAb to 24 different human TCRVβ chains covering approximately 70% of the known human TCRVβ repertoire (IOTest Beta Mark mAb kit; Beckman Coulter, Marseille, France) combined with mAb to CD3, CD4 and CD8. TCRVβ usage of autoBLCL-specific T cells in WML-TCL was identified by CD3, CD4, CD8 and TCRVβ combined with intracellular IFNγ staining and flow cytometry. T-cell clonality was also determined by the TCR gamma (TCRγ) gene rearrangement Assay 2.0 (Invivoscribe, San Diego, CA) performed in duplo on DNA isolated from FACS-sorted viable CD8^+^ T cells of paired brain tissue-derived TCL of eight MS patients, and as control PBMC and two monoclonal human T-cell leukemic cell lines (MOL3 and KL 1985-001), according to manufacturers’ instructions (Invivoscribe).

### Data analysis and statistics

Flow cytometry data were analyzed with FlowJo (Tree Star Inc., Ashland, OR) software. Paired data were assessed using Wilcoxon-signed rank test. Significance of variation in cMSAg- and autoBLCL-specific T-cell reactivity was determined by ANOVA for CD4^+^ and CD8^+^ T cells separately. Unpaired t-test was used to determine significance of autoBLCL T-cell reactivity. All statistical analyses were performed using Graphpad Prism 5 software (Graph Pad Inc., La Jolla, CA).

## Results

### Macroscopically defined NAWM of MS patients with advanced disease contain diffuse white matter abnormalities

**Fig. 1:**
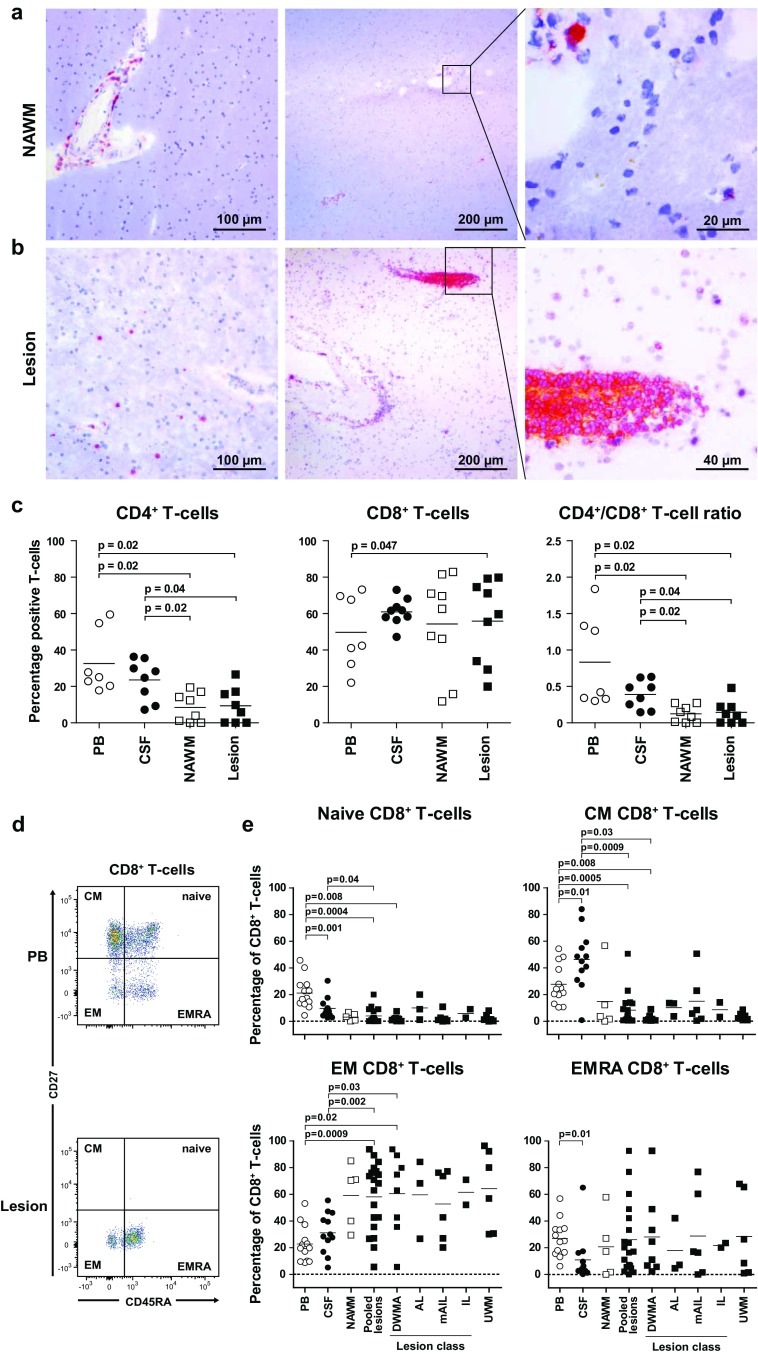
CD8^+^ T cells in normal-appearing and diseased white matter tissues of MS patients preferentially express an effector memory phenotype. **a**, **b** Parenchymal T cells are selectively detected in active MS lesions. 10-µm cryostat sections of paired (**a**) normal-appearing white matter (NAWM) and (**b**) white matter lesion (WML) of two representative patients of five MS patients analyzed. CD3 expressing cells (T cells) were stained with 3-amino-9-ethylcarbazole (*red color*) and counterstained with hematoxylin (*blue color*). Whereas perivascular T cells were detected in both NAWM and WML, parenchymal T cells were exclusively detected in active WML of MS patients. **c** Percentages of CD4^+^ and CD8^+^ T cells, and CD4^+^/CD8^+^ T-cell ratio, are shown for paired PB (*open circles*), CSF (*closed circles*) and histologically defined as NAWM (*open squares*) and WML (*filled squares*). Lymphocytes were isolated from paired peripheral blood (PB), cerebrospinal fluid (CSF), NAWM and WML (lesion) from patients with advanced MS (*n* = 17) and subjected to multiplex flow cytometry. Gating procedure of CD4^+^ and CD8^+^ T cells is shown in Online Resource 3. **d** CD8^+^ T cells were subdivided in naïve (CD27^+^CD45RA^+^), central memory (CM; CD27^+^CD45RA^−^), effector memory (EM; CD27^−^CD45RA^−^) and terminally differentiated effector memory (EMRA; CD27^−^CD45RA^+^) T cells. Gating procedure is shown for representative paired PB and WML-derived CD8^+^ T cells. **e** The frequency of naïve, CM, EM and EMRA CD8^+^ T cells is shown for paired PB, CSF and white matter brain tissues that were immunohistologically classified as NAWM, diffuse white matter abnormalities (DWMA), active lesions (AL), mixed active/inactive lesions (mIAL), inactive lesion (IL) or unconfirmed white matter tissues (UWM) (see Online Resource 3 for criteria applied for MS WM classification). *Horizontal lines* represent the mean frequencies. Wilcoxon matched pairs test was used to calculate significance

To characterize the role of T cells in MS, we collected paired PB, CSF, NAWM and WML specimens from 27 deceased patients with advanced MS (Online Resource 1). The presence and classification of the WM tissues sampled was determined by IHC on representative snap-frozen sections of surplus NAWM and WML sections (Online Resource 3) [29]; provided adequate size of brain tissues was available. All macroscopically defined lesions were confirmed as WML and all contained demyelinated areas classified as AL, mAIL or IL [29]. Notably, 9 of 20 (45%) macroscopically defined NAWM tissues presented as DWMA consisting of increased numbers of macrophage/microglia and high HLA class II expression (Online Resource 1). These DWMA represent periplaque abnormalities, Wallerian degeneration or pre-lesional changes [29]. The spatial distribution of T cells was determined on paired NAWM and WML tissues from five MS patients. In both NAWM and active WML, T cells were mainly detected in the Virchow–Robin space (Fig. 1a). However, parenchyma-infiltrating T cells were selectively observed in WML suggesting that these T cells are not part of immune surveillance, but rather involved in the local inflammatory response [41, 56].

### Effector memory CD8^+^ T cells are the main T-cell subset in NAWM and WML of MS patients

To determine the phenotype and differentiation status of T cells in MS patients ex vivo, paired PB, CSF, and lymphocyte-enriched NAWM- and WML-derived single cell suspensions of 17 MS patients were subjected to multiplex flow cytometric analysis. T cells were selected by lymphocyte, CD45^high^ and CD3 gating, and finally sub-classified as CD3^+^CD4^high^ and CD3^+^CD8^high^ T cells based on PB-derived T cells. In contrast to PB and CSF, brain tissue-derived CD3^+^ cells frequently expressed low levels of CD4 or CD8, and on occasion were devoid of CD4 and CD8 expression (Online Resource 4). As these different T-cell subtypes are difficult to differentiate by flow cytometry and no consensus exists in literature on their origin, we omitted them from further analysis and only focused on T cells expressing high levels of CD4 or CD8. The latter uniform gating strategy was chosen to compare the activation and differentiation status of the same CD4^+^ and CD8^+^ T cells between multiple anatomic locations of the same individual. In both NAWM and WML, CD8^+^ T cells dominated as shown by the significantly lower CD4^+^/CD8^+^ T-cell ratio in NAWM and WML compared to PB and CSF (Fig. 1c).

Next, the differentiation status of T cells was compared between compartments based on differential surface expression of CD45RA and CD27 (Fig. 1c) [10]. Naive (T_NA_; CD27^+^CD45RA^+^) CD8^+^ T cells were readily identified in PB, less frequently in CSF and rarely in NAWM and WML (Fig. 1d). Central memory (T_CM_; CD27^+^CD45RA^−^) CD8^+^ T cells were the dominant phenotype in CSF. Effector memory (T_EM_; CD27^−^CD45RA^−^) CD8^+^ T cells predominated in both NAWM and WML, with frequencies twofold higher compared to PB and CSF. Finally, terminally differentiated memory (T_EMRA_; CD27^−^CD45RA^+^) T-cell frequencies were equivalent in PB, NAWM and WML, but lower in CSF. No significant differences in CD4^+^/CD8^+^ T-cell ratio and CD8^+^ T-cell differentiation status were observed between different WM types. Low numbers of CD4^+^ T cells in most WML and NAWM specimens precluded conclusive definition of their differentiation status (data not shown). In conclusion, CD8^+^ T_EM_ cells are enriched in both NAWM and WML of the MS patients analyzed.

**Fig. 2:**
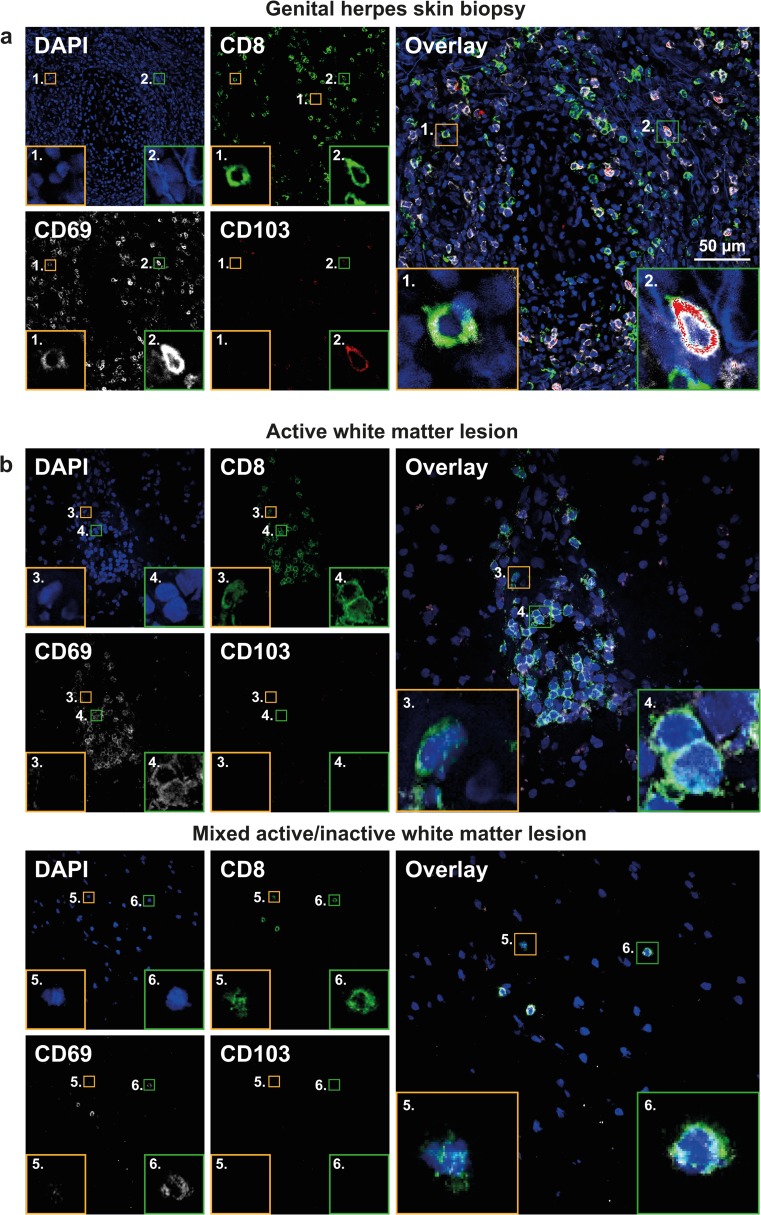
CD8^+^ T cells in white matter lesions of MS patients preferentially express CD69, but not CD103. **a**, **b** Representative triple immunofluorescent stainings on 8-μm cryosections of **a** skin biopsies from six genital herpes patients and **b** immunohistochemically classified white matter lesions (WML) of six MS patients. Tissues were stained for CD8 (*green color*), CD69 (*white color*) and CD103 (*red color*) using specific monoclonal antibodies (mAbs) and isotype specific fluorochrome-conjugated secondary antibodies. Sections were counterstained with 4′,6-diamidino-2-phenylindole (DAPI; *blue color*) and analyzed using a Zeiss LSM-700 confocal laser microscopy and ZEN software. Skin biopsies of genital herpes patients were stained as positive control to validate staining strategy by confirming localization tissue-resident CD8^+^ T cells based on differential CD69 and CD103 staining: CD8^+^CD69^+^CD103^−^ (**a**; *inset 1*) and CD8^+^CD69^+^CD103^+^ T cells (**a**; *inset 2*). **b** In WML of MS patients, perivascular (*top panels*) and parenchymal CD8^+^ T cells (*bottom panels*) were incidentally CD69^−^CD103^−^ T cells (**b**; *insets 3 and 5*) and predominantly CD69^+^CD103^−^ T cells (**b**; *insets 4 and 6*). *Size bar* is indicated in *top-right image*

The expression of CD69 and CD103, two proteins described to enhance long-term retention of T cells within peripheral tissues also referred as T_RM_ cells [51], was determined on CD8^+^ T cells in WML cryosections of six MS patients by triple immunofluorescence staining. We first validated our staining protocol by analyzing skin biopsies of 6 HSV-2 GH patients. In agreement with a previous study, the majority of human genital skin CD8^+^ T_RM_ expressed CD69 and about 20% of these cells co-expressed CD103 (Fig. 2a) [46, 51]. In contrast, both perivascular and parenchymal CD8^+^ T cells in WML of MS patients exclusively expressed the CD8^+^CD69^+^CD103^−^ phenotype (Fig. 2b). These data contrast previous studies on mouse CNS- and human glioma-derived CD8^+^ T cells showing that about half of CD8^+^CD69^+^ T_RM_ cells in CNS tissue co-express CD103 [27]. Because CD69 is also expressed by T cells early after TCR and cytokine activation [9], and since CD103 is debated as reliable T_RM_ marker [34], the CD8^+^CD69^+^CD103^−^ T cells detected in WML of the MS patients analyzed may denote genuine CD8^+^ T_RM_ cells, represent activated T cells or a mixture thereof [10, 27, 34, 51].

### Phenotype of CD8^+^ T cells in white matter lesions of MS patients implicate a local antigen-specific cytotoxic T-cell function

**Fig. 3:**
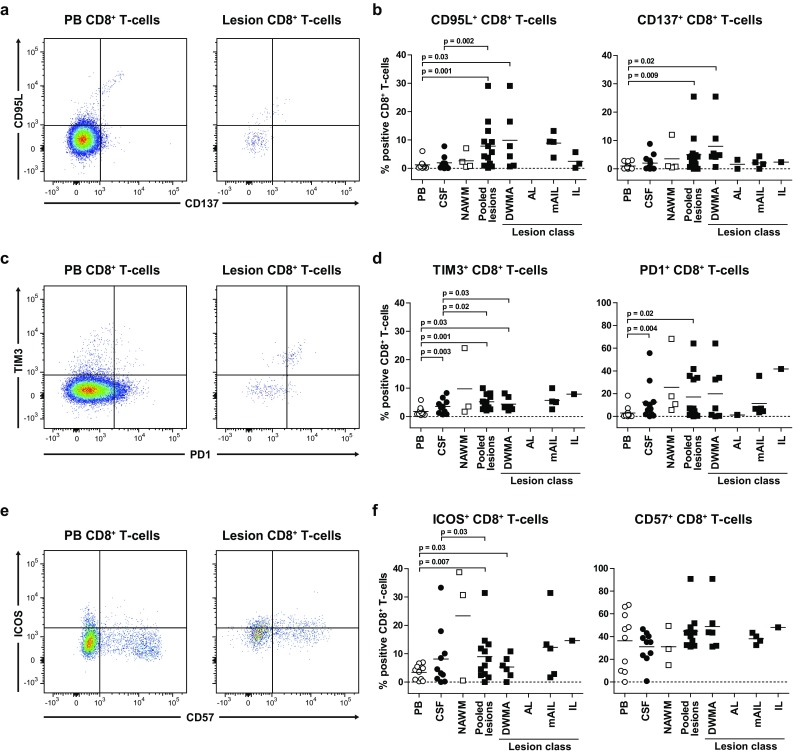
CD8^+^ T cells in white matter lesions of MS patients are chronically activated T cells expressing a cytotoxic effector T-cell phenotype. Flow cytometric analysis of cytotoxic molecule CD95L and co-stimulatory receptor CD137 (**a**, **b**), co-inhibitory receptors TIM3 and PD1 (**c**, **d**) and co-stimulatory molecule ICOS and senescence marker CD57 (**e**, **f**) expression on CD8^+^ T cells in paired peripheral blood (PB, *open circles*), cerebrospinal fluid (CSF, *closed circles*) and brain tissues histologically defined as normal-appearing white matter (NAWM, *open squares*) and white matter lesions (WML, *filled squares*) classified as diffuse white matter abnormalities (DWMA), active lesions (AL), mixed active/inactive lesions (mIAL) and inactive lesions (IL) of 17 MS patients (see Online Resource 3 for criteria applied for MS WM classification). Lymphocyte gating was performed as described in the legend of Online Resource 4. Gating strategies and percentages of marker positive CD8^+^ T cells in paired anatomic compartments are shown. If a parent population contained <100 events, daughter populations were omitted in further analysis. *Horizontal lines* indicate the mean. Wilcoxon matched pairs test was used to calculate significance

To determine the effector function and activation status of CD8^+^ T cells ex vivo between different compartments of MS patients, expression of the death signal and co-stimulatory receptor FasL (CD95L) and the early activation marker CD137 were determined by multiplex flow cytometry on CD8^+^ T cells (Fig. 3a). Expression of both CD95L and CD137 is induced on antigen-stimulated CD8^+^ T cells and interaction of FasL with its receptor Fas may lead to target cell lysis [10]. Compared to PB, significantly more CD8^+^ T cells in WML were positive for CD95L and CD137, which was predominantly observed in DWMA (Fig. 3b).

**Fig. 4:**
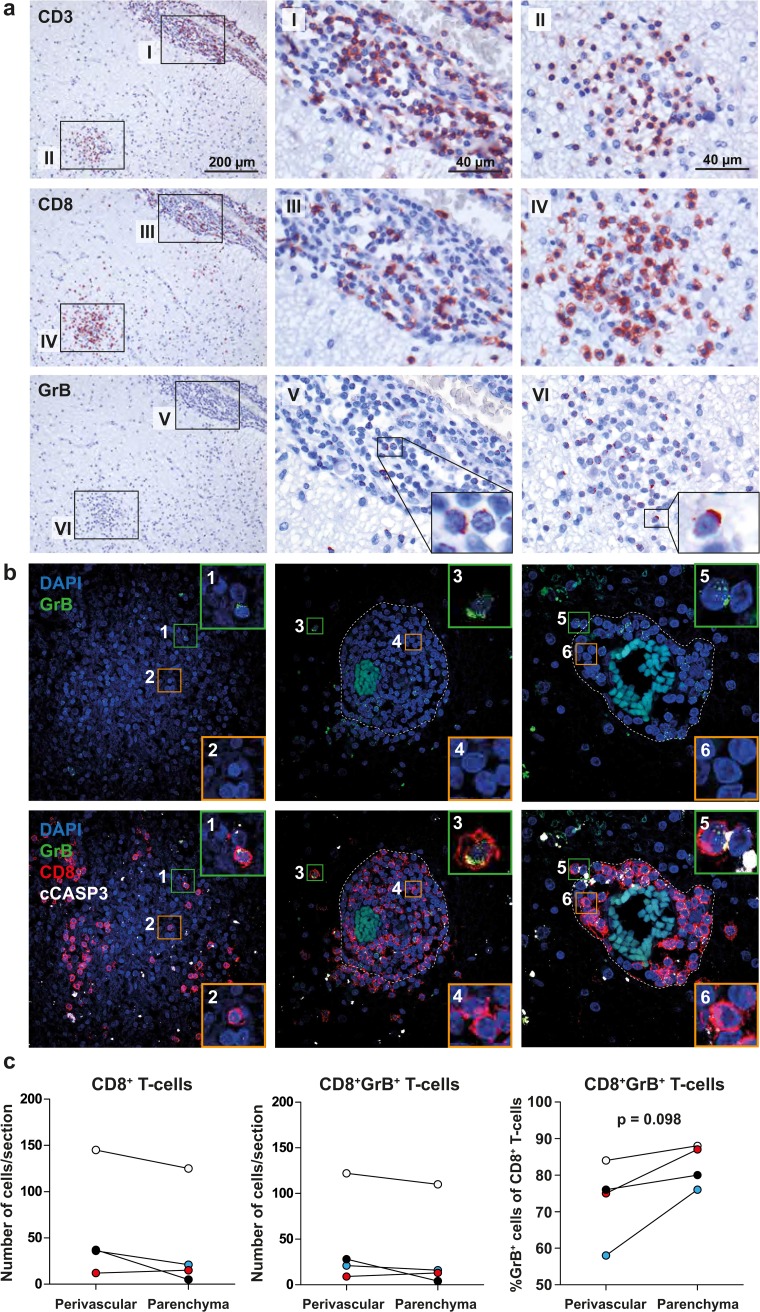
CD8^+^ T cells in white matter lesions of MS patients express granzyme B. **a** Representative stainings on 6-µm sections of a formalin-fixed and paraffin-embedded (FFPE) mixed active/inactive white matter lesion (mAIL) of one of four MS patients analyzed. CD3 (*top panel*), CD8 (*middle panel*) and granzyme B (grB) expressing cells (*bottom panel*) were stained with 3-amino-9-ethylcarbazole (*red color*) and counterstained with hematoxylin (*blue color*). Abundant punctate expression of grB was detected in perivascular (*insets I, III and V*) and parenchymal (*insets II, IV and VI*) CD8^+^ T cells. Granzyme B polarization was observed in both perivascular and parenchymal CD8^+^ T cells (*insets V and VI*). **b** Representative maximum intensity projections of z-stack laser confocal microscopy images of immunofluorescent triple stainings for grB (*green color*), CD8 (*red color*) and the early apoptotic cell marker “cleaved caspase-3” (cCASP3; *white color*). Stained sections were counterstained with DAPI (*blue color*). Representative stainings of three mAIL are shown of 12 immunohistochemically classified WML tissues of 10 MS patients analyzed. Punctated (*inset 1*) and polarized grB expression by CD8^+^ T cells (*insets 3 and 5*), as well as grB-negative CD8^+^ T cells are shown (*insets 2*, *4 and 6*). Co-localization of grB and cCASP3 is observed in a cell adjacent to a CD8^+^ T-cell with polarized grB suggesting grB-mediated killing of the respective target cell (*inset 5*). *Dotted line* represents the glia limitans separating the perivascular space and parenchyma. **c** The grB-expressing CD8^+^ T cells were counted in the perivascular space and parenchyma of mAIL of four MS patients analyzed. Wilcoxon matched pairs test was used to calculate significance

In addition to the Fas/FasL-pathway, T-cell cytotoxicity involves exocytosis of granules containing perforin and granzymes [38]. Previous studies showed lowered [56] or absent [30] intrathecal expression of perforin and grB by CD8^+^ T cells in non-inflammatory CNS conditions, suggesting a functionally repressed phenotype of CNS surveilling T cells. Contrastingly, grB^+^CD8^+^ T_EM_ cells appeared enriched in CSF of MS patients [22]. To address this issue, consecutive sections of mAIL WML from four MS patients were stained for CD3, CD8 and grB (Fig. 4a). Whereas the majority of CD8^+^ T cells showed abundant whilst punctated grB expression, a fraction of CD8^+^ T cells showed a more polarized grB expression pattern in both perivascular and parenchymal CD8^+^ T cells (Fig. 4a). GrB distribution and cytotoxic potential of CD8^+^ T cells was confirmed using z-stack confocal laser microscopy on 12 WML biopsies of 10 additional MS patients assayed by triple immunofluorescence staining for CD8, grB and the early apoptotic marker “cleaved caspase 3” (cCASP3). Occasionally adjacent cells expressed cCASP3 suggesting CD8^+^ T-cell-mediated cytotoxicity in situ (Fig. 4b). Parenchymal CD8^+^ T cells were most frequently identified in mAIL (data not shown). The frequency of grB-expressing CD8^+^ T cells in the parenchyma tended to be higher compared to the paired perivascular regions in mAIL (Fig. 4c). Collectively, the data indicate that CD8^+^ T cells in WML of MS patients analyzed express a cytotoxic phenotype indicative for T cells recognizing their cognate antigen in situ [38].

### CD8^+^ T cells in white matter lesions of MS patients are chronically activated T cells expressing a cytotoxic effector T-cell phenotype

The repertoire of co-stimulatory and co-inhibitory receptors determines the fate of T cells after antigenic challenge [10, 58]. Chronic antigenic stimulation leads to upregulation of co-inhibitory receptors that negatively affect their proliferative potential, effector function and survival [10]. In CNS, co-signaling via transmembrane immunoglobulin mucin 3 (TIM3) and programmed cell death type 1 (PD1) are important immunoregulatory mechanisms [25]. Hence, the frequency of CD8^+^ T cells expressing TIM3 and PD1 ex vivo was compared between different compartments by multiplex flow cytometry (Fig. 3c, d). Compared to PB, significantly more CD8^+^ T cells in both CSF and MS lesions expressed TIM3 and PD1. In WML, this was largely attributed to CD8^+^ T cells in DWMA. Chronic T-cell stimulation may ultimately result in an apoptosis-resistant and senescent phenotype, characterized by human natural killer-1 receptor expression (CD57). This phenotype is potentially reverted by specific cytokines (e.g., IL-2 and IL-15) resulting in upregulated expression of the inducible co-stimulator (ICOS) molecule on T cells [58]. No significant difference was observed in ex vivo CD57 expression by CD8^+^ T cells between different compartments, arguing against senescent CD8^+^ T cells in MS (Fig. 3e, f). Frequencies of ICOS^+^CD8^+^ T cells were significantly elevated in WML compared to PB and CSF, but were similar between NAWM and the different types of WML. Collectively, the data demonstrate that CD8^+^ T cells in WML of MS patients exhibit characteristics of chronically activated T cells.

**Fig. 5:**
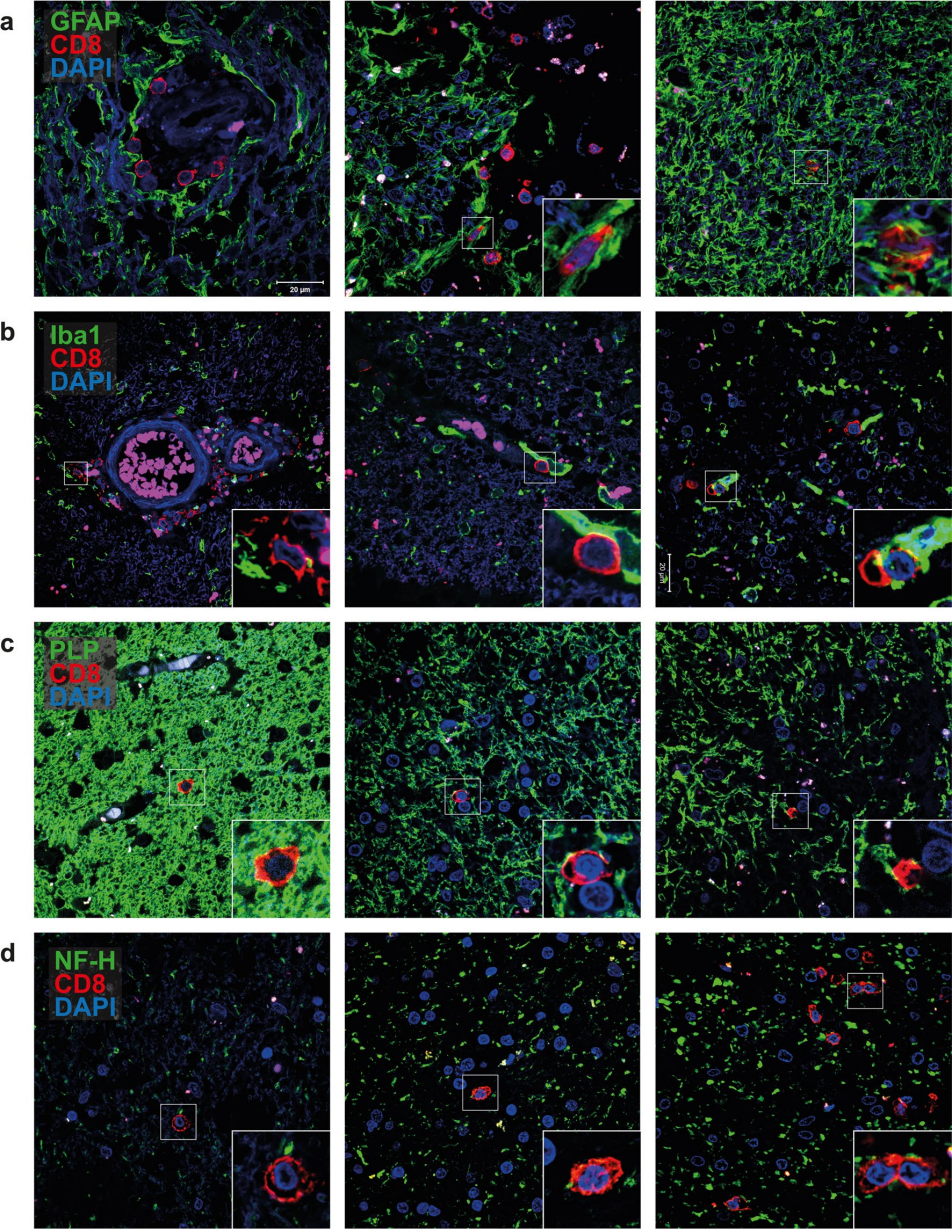
CD8^+^ T cells interact with all major brain-resident cell types in white matter lesions of MS patients. Representative double-immunofluorescence stainings on 8-μm sections of 12 formalin-fixed paraffin-embedded white matter lesion (WML) tissues from 10 MS patients are shown for **a** glial fibrillary acidic protein (GFAP: marker for astrocytes); **b** ionized calcium-binding adapter molecule 1 (Iba1: marker for microglia); **c** proteolipid protein (PLP: marker for oligodendrocytes) and **d** neurofilament heavy chain (NF-H: marker for neurons; *all green color*) combined with CD8 (*red color*), counterstained with 4′,6-diamidino-2-phenylindole (DAPI; *blue color*) and finally analyzed using a Zeiss LSM-700 confocal laser microscopy and ZEN software. Perivascular CD8^+^ T cells interact with astrocytes (**a** *left* and *middle panel*) and microglia (**b** *left* and *middle panel*). Parenchymal CD8^+^ T cells also interact with astrocytes (**a** *right panel*), microglia (**b** *right panel*) and oligodendrocytes (**c**) in fully myelinated (*left panel*) and partially demyelinated areas (*middle* and *right panels*). Parenchymal CD8^+^ T cells also interact with neurons (**d**) in areas without (*left panel*), with moderate (*middle panel*) and with prominent axonal swelling (*right panel*). The latter is indicative of axonal damage. *Insets* show specific interactions between CD8^+^ T cells and the major brain-resident cells analyzed for. *Scale bar* is indicated (**a**, *top left panel*)

To identify the brain cell types that interact with CD8^+^ T cells, double-immunofluorescence stainings on WML tissue sections of 12 FFPE tissues of 10 MS patients were performed (Online Resource 2). Hereto, anti-CD8 mAb was combined with antibodies directed to specific proteins expressed by human astrocytes (GFAP), microglia (Iba1), oligodendrocytes (PLP) and neurons (NF-H) (Fig. 5). Perivascular CD8^+^ T cells interacted preferentially with astrocytes and microglia. Parenchymal CD8^+^ T cells interacted with astrocytes, microglia, oligodendrocytes in fully myelinated and partially demyelinated areas. Moreover, they interacted with neurons in areas with and without prominent axonal swelling, indicative of neuronal stress or axonal damage due to demyelination [6]. Hence, CD8^+^ T cells in WML of the MS patients analyzed showed no preferential interaction with a specific brain cell type (Fig. 5).

### Correlation of TCR repertoire between TCL generated from distinct lesions of the same MS patient

The markers expressed by CD8^+^ T cells in WML suggest antigen-driven activation and potentially retention of specific TCC in affected tissue of MS patients (Figs. 3, 4). Given the low T-cell numbers in the clinical specimens obtained, T-cell clonality was assayed on short-term TCL generated by non-specific stimulation of T cells recovered from paired CSF, NAWM and WML specimens. TCRVβ chain usage of CD4^+^ and CD8^+^ T cells was determined by flow cytometry on paired NAWM- and WML-TCL (*n* = 4 patients) and paired WML-TCL (*n* = 6 patients) (Online Resource 5). Most TCL showed oligoclonal TCRVβ repertoires, but no specific TCRVβ predominated among patients. Whereas the TCRVβ repertoire of paired NAWM- and WML-TCL did not match, a significant correlation was observed for CD4^+^ and particularly CD8^+^ T cells in paired WML-TCL of two and four of six MS patients, respectively (Online Resource 5). The shared TCRVβ chain usage implicates enrichment of specific TCC in distinct WML of the same MS patient. To address this issue in more detail, we assayed the composition of the TCR repertoire of CD8^+^ T cells in paired NAWM/WML- (*n* = 3) and WML/WML-TCL (*n* = 5) by performing TCRγ-rearrangement spectratyping on sorted CD8^+^ T cells (Online Resource 6) [13]. The TCRG locus is rearranged early during T-cell development in both TCR-αβ and -γδ lineage precursors and is considered the prototypic TCR locus for the detection of T-cell clonality in clinical specimens [13]. Typical polyclonal Gaussian curves signifying polyclonality, or one or two peaks illustrative for the rearrangement of the TCRG locus on one or both chromosomes, were detected when human PBMC or monoclonal T-cell leukemic cell lines (MOL3 and KL 1985-001) were assayed, respectively (Online Resource 6) [13]. TCRG GeneScan analyses demonstrated a reproducible, markedly restricted pattern of TCRG rearrangements by CD8^+^ T cells in the brain-derived TCL analyzed. These data confirm that the oligoclonal CD8^+^ T-cell populations in TCL generated from paired NAWM and WML of the same MS patient were clonally distinct (patients #2, #4 and #8; Online Resource 6), whereas CD8^+^ T cells in paired WML-TCL of multiple MS patients showed a strong clonal overlap (patients #5, #6, #7 and #9; Online Resource 6). These data suggest that the paired WML sampled contained identical CD8^+^ TCC potentially involved in the disease process.

### No substantial T-cell responses towards human candidate MS-associated autoantigens in T-cell lines generated from cerebrospinal fluid and white matter of MS patients

Previous studies, both on EAE and MS patients, support a key role of pathogenic T cells directed to various cMSAg including glia- (KIR4.1, S100B), oligodendrocyte- (MAG, MBP, MOG) and neuron-specific proteins (CNTN2 and NFASC) [17–19]. However, autoreactive T cells are also a physiological component of the healthy immune system making disease association difficult [17–19]. Among the various genetic predictors of MS development, three specific HLA-I and -II alleles have been identified: HLA-A*0301, -DRB1*1501 and -DRB*1301 [49]. Their association with MS may involve presentation of specific cMSAg-derived peptides that trigger pathogenic T-cell responses [44].

**Fig. 6:**
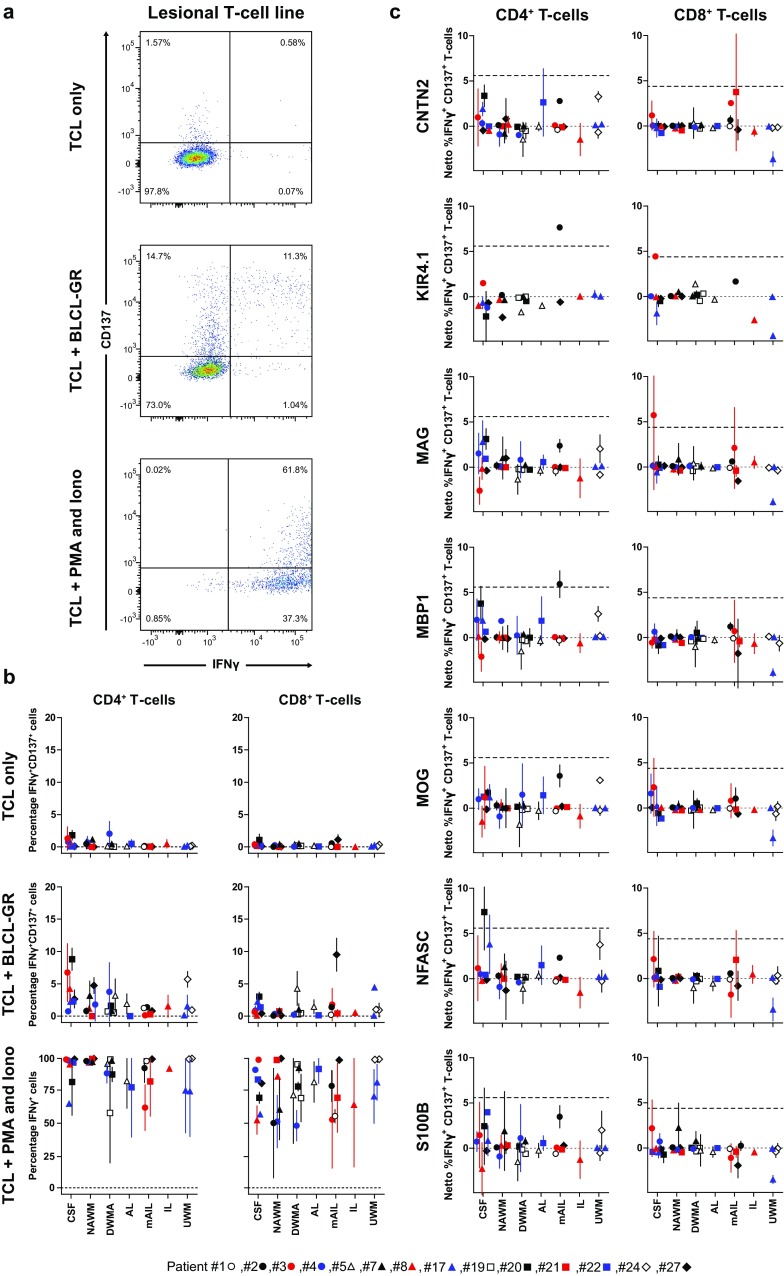
T-cell lines generated from paired cerebrospinal fluid and white matter brain tissue of MS patients show no substantial T-cell reactivity towards candidate human MS-associated autoantigens. Short-term T-cell lines (TCL) were generated by non-specific stimulation of T cells recovered from paired cerebrospinal fluid (CSF) and white matter brain tissues from 14 MS patients, which were immunohistologically classified as normal-appearing white matter (NAWM), diffuse white matter abnormalities (DWMA), active lesions (AL), mixed active/inactive lesions (mAIL), inactive lesions (IL) or undefined white matter tissue (UWM) (see Online Resource 3 for criteria applied for MS WM classification). An HLA-matched Epstein–Barr virus-transformed B-cell line (i.e., BLCL-GR) was used to assay T-cell reactivity towards candidate human MS-associated autoantigens (cMSAg). **a** Antigen-specific T cells were enumerated by determining co-expression of intracellular interferon gamma (IFNγ) and CD137 using multiplex flow cytometry. Gated CD8^+^ T cells from mAIL-derived TCL of MS patient #27 (see Online Resource 1) is representatively shown. CD8^+^ T cells alone (*top panel*), stimulated with untransduced BLCL-GR (*middle panel*) or with phorbol myristate-acetate (PMA) and ionomycin (Iono) are shown. **b** The frequency of IFNγ and CD137 co-expressing CD4^+^ (*left panel*) and CD8^+^ T cells (*right panels*) that were cultured alone (*top panels*) or co-cultured with untransduced BLCL-GR (*middle panels*) are shown. The *bottom panel* shows the frequency of IFNγ-expressing CD4^+^ (*left panel*) and CD8^+^ T cells (*right panel*) after stimulation with a cocktail of T-cell mitogens (i.e., PMA and Iono). **c** BLCL-GR were nucleofected with human candidate MS autoantigens (cMSAg) expression vectors encoding human contactin-2 (CNTN2), inwards rectifying potassium channel (KIR4.1), myelin-associated glycoprotein (MAG), myelin basic protein isoform 1 (MBP1), myelin oligodendrocyte glycoprotein (MOG), neurofascin (NFASC) or S100 calcium-binding protein B (S100B). TCL were co-cultured with the respective cMSAg-expressing BLCL-GR and the phenotype and frequency of cMSAg-specific T cells determined by flow cytometry. The netto frequency of cMSAg-specific T cells, corrected for reactivity towards untransduced BLCL-GR, is shown as the percentage IFNγ^+^CD137^+^ CD4^+^ (*left panel*) and CD8^+^ T cells (*right panel*). *Symbols* represent the individual MS patients analyzed (*n* = 14; specified at the *bottom of the figure*). The majority of TCL were assayed at least two times, of which *vertical lines* represent the mean and standard deviation. *Horizontal dashed lines* depict the cut-off for positive calls for CD4^+^ and CD8^+^ T cells, allowing a 0.1% false discovery. Significance of variation in cMSAg-specific T-cell reactivity was determined by ANOVA for CD4^+^ and CD8^+^ T cells separately

To test this hypothesis, CD4^+^ and CD8^+^ T-cell reactivity towards these seven human cMSAg was determined for TCL of MS patients (*n* = 14) carrying the three MS-associated HLA risk alleles (Online Resource 1). Hereto, an allogeneic BLCL (BLCL-GR), matched for these three HLA alleles, was transduced and selected in vitro to express the individual cMSAg, and measles virus fusion protein as control T-cell antigen, constitutively at high level (data not shown) [42]. Because no human cMSAg-specific T-cell clone or lines were available, we used MVF-transduced BLCL-GR as APC to validate the applicability of our expression system to process and present endogenously expressed antigens by both HLA-I and II. As shown previously, both the MVF-specific CD4^+^ (4-F99) and CD8^+^ TCC (2-F40) recognized MVF-transduced GR-BLCL efficiently (data not shown) [42]. Next, human cMSAg-specific T-cell reactivity was determined by multiplex flow cytometry on CSF-, NAWM- and WML-TCL of HLA-matched MS patients using the cMSAg-transduced BLCL-GR as antigen presenting cells (Fig. 6). Whereas TCL only and TCL co-cultured with untransduced BLCL-GR showed limited T-cell responses, TCL stimulated with a cocktail of the T-cell mitogens PMA and Iono showed a uniformly high expression of the T-cell activation marker IFNγ demonstrating that the T cells in the TCL were immunocompetent and not exhausted (Fig. 6a, b).

The BLCL-GR-reactive CD4^+^ (mean ± SD 2.1% ± 2.4) and CD8^+^ (1.3 ± 2.0%) T-cell responses of the CSF- and brain tissue-derived TCL were low and the inter-assay variation was 1.69% for CD4^+^ T cells and 1.44% for CD8^+^ T cells in independent replicate experiments. Consequently, the threshold for positive calls was 5.6% for CD4^+^ T cells and 4.4% for CD8^+^ T cells allowing a one-tailed 0.1% false discovery rate [42]. Marginal cMSAg-specific T-cell responses were detected in only 4 of 207 (1.9%) cMSAg/TCL-combinations, including two CSF-TCL (Fig. 6c). Overall, the limited cMSAg-specific T-cell reactivity in paired CSF-, NAWM- and WML-TCL argues against their involvement in the perpetuation of disease in the 14 MS patients analyzed.

### T cells recovered from white matter lesions of MS patients recognize autologous Epstein–Barr virus-infected B cells

**Fig. 7:**
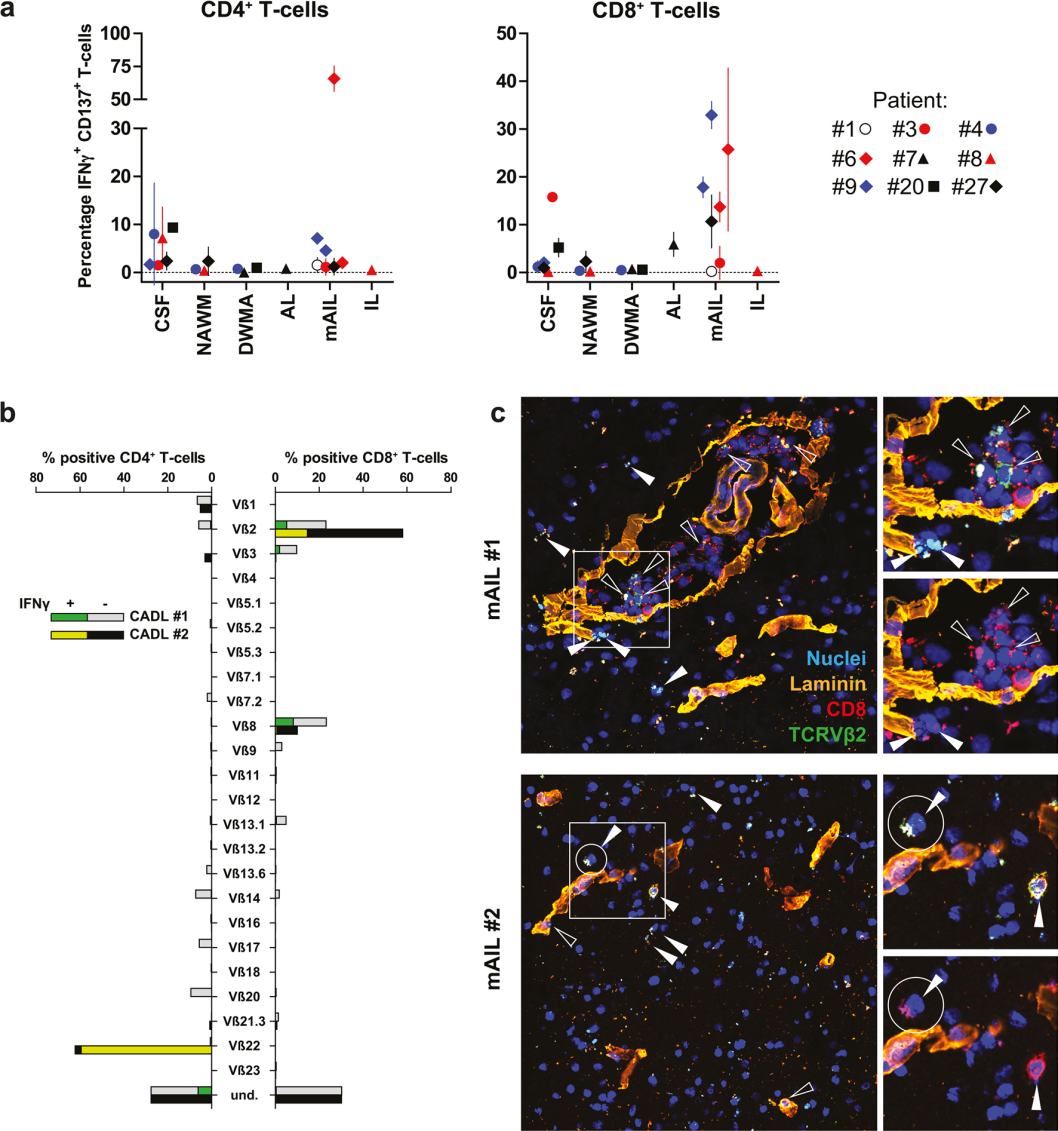
White matter lesion-derived CD8^+^ T cells recognize autologous Epstein–Barr virus-transformed B cells and localize in the parenchyma to form immune synapses. **a** Short-term T-cell lines (TCL) were generated by non-specific stimulation of T-cell recovered from paired cerebrospinal fluid (CSF) and white matter brain tissues from nine MS patients, which were immunohistologically classified defined as normal-appearing white matter (NAWM), diffuse white matter abnormalities (DWMA), active lesions (AL), mixed active/inactive lesions (mAIL) and inactive lesions (IL) (see Online Resource 3 for criteria applied for MS WM classification). The TCL were incubated with autologous Epstein–Barr virus-transformed B cell lines (autoBLCL). Next, the phenotype and frequency of autoBLCL-specific T cells was determined by co-expression of intracellular interferon gamma (IFNγ) and CD137 using multiplex flow cytometry. The frequency of autoBLCL-reactive T cells is shown as the percentage of IFNγ^+^CD137^+^ CD4^+^ (*left panel*) and CD8^+^ T cells (*right panel*). *Symbols* represent individual donors (*n* = 9; specified in the legend) and vertical lines represent the mean and standard deviation of at least two independent experiments per TCL. Significance of variation in autoBLCL T-cell reactivity was determined by ANOVA for CD4^+^ and CD8^+^ T cells separately. **b** TCL generated from two anatomically distinct mAIL of MS patient #6 (see Online Resource 1) were cultured with autoBLCL to assay the T-cell receptor variable β chain (TCRVβ) usage of the reactive T cells, determined by intracellular interferon gamma (IFNγ) expression, using multiplex flow cytometry. The frequency of CD4^+^ (*left x*-axis) and CD8^+^ T cells (*right x*-axis) of specific TCRVβ families (*y*-axis) are depicted (*gray bars* lesion #1, *black bars* lesion #2). The frequency of autoBLCL-reactive CD4^+^ T cells and CD8^+^ T cells of each TCRVβ family is shown (*stacked green bars* IFNγ^+^ T cells). Results shown are representative for two independent experiments. “Und.” refers to T cells expressing a TCRVβ chain not covered by the TCRVβ-family-specific monoclonal antibody panel used. **c** Triple immunofluorescence staining for TCRVβ2 (*green color*), laminin (*orange color*) and CD8 (*red color*) in surplus WML tissue sections (8 µm) containing WML #1 and #2, from which the corresponding TCLs shown in panel “A” were generated. Nuclei were stained with DAPI (*blue color*). TCRVβ2^+^ CD8^+^ T cells reside in the perivascular cuff (*open arrowhead*) and the parenchyma (*closed arrowhead*) of both distinct WML of the same patient. The majority of parenchymal T cells show polarization of both CD8 and TCRVβ2 (*encircled cells in top-right insets*). Images of representative stainings are shown

Epstein–Barr virus infection is a major environmental risk factor to develop MS in genetically predisposed individuals [33, 49]. Recent data argue for a role of EBV-specific T cells in MS pathology [2, 32, 43], but proof of their presence in brain tissues of MS patients is lacking. This prompted us to investigate T-cell responses towards autoBLCL in paired CSF-, NAWM- and WML-TCL. Concordant with earlier studies, about 5% of autoBLCL cells expressed the late lytic viral antigen glycoprotein 350, demonstrating that these cells have spontaneously entered the lytic cycle and most likely expressed the whole EBV proteome (Online Resource 7). Accordingly, autoBLCL were considered appropriate APC to analyze EBV-specific T-cell reactivity [1, 43]. Due to limited cell numbers and viability, autoBLCL could only be generated for nine MS patients. In contrast to NAWM- and DWMA-TCL, substantial T-cell responses towards autoBLCL, predominantly CD8^+^ T cells, were detected in multiple mAIL- and the sole AL-TCL analyzed. This response was most pronounced among IAL-TCL: five of seven IAL-TCL assayed had brisk T-cell responses towards autoBLCL-specific (Fig. 7a). Notably, these strong CD8^+^ T-cell responses correlated between paired TCL generated from distinct mAIL of the same patient: MS patients #6 and #9 (Fig. 7a). The data implicate enrichment of autoBLCL-reactive CD8^+^ T cells in AL and mAIL of MS patients that are potentially involved in the disease process [57].

These autoBLCL-reactive T cells are potentially enriched in brain tissue due to local stimulation by EBV proteins [53]. Hence, we determined the presence of the EBV transcript EBER1, which is highly expressed during both latent and lytic EBV infection [59], in surplus brain tissue from which the respective NAWM- and WML-TCL were generated. No EBER1 RNA was detected implicating that productive EBV infection or the presence of latently EBV-infected B cells in the brain tissues sampled is highly unlikely (data not shown).

### Clonally expanded autologous BLCL reactive CD8^+^ T cells are located in brain parenchyma and form immune synapses

The combined data on congruent TCR clonality, selective TCRVβ expression by CD8^+^ T-cell showing reactivity towards autoBLCL in paired intra-lesional TCL suggest local enrichment of the same CD8^+^ TCC. Among the high autoBLCL T-cell responders, sufficient T-cell quantities were only available for the mAIL-TCLs of MS patient #6 for follow-up studies. TCRVβ analysis revealed that TCRVβ2^+^ T cells dominated the oligoclonal CD8^+^ T-cell response towards autoBLCL in the paired intra-lesional TCL of MS patient #6: mAIL-TCL#1 (32%) and #2 (89%) (Fig. 7b). Furthermore, the autoBLCL-reactive CD4^+^ T-cell response in mAIL-TCL#2 was restricted to TCRVβ22 (99%) expressing T cells. Next, we localized TCRVβ2^+^CD8^+^ T cells, as proxy for the dominant autoBLCL-reactive CD8^+^ TCC, by IHC in the respective WML tissues. The TCRVβ2^+^CD8^+^ T cells were detected in the Virchow–Robin space and parenchyma in both mAIL of patient #6 (Fig. 7c). Notably, the majority of parenchymal TCRVβ2^+^CD8^+^ T cells showed polarization of both TCR and CD8, indicative for immune synapse formation and confirmed by Z-stack analysis (Online Resource 8). Collectively, the data suggest the involvement of CD8^+^ T cells recognizing antigens expressed by autoBLCL, and potentially the same TCC, in the inflammatory process in two distinct mAIL of the same MS patient.

## Discussion

The current study provides novel insights into the phenotypic and functional characteristics of the T-cell response in CSF and (non-)affected brain tissue of MS patients. Three main findings are reported. First, T cells in MS lesions are predominantly CD8^+^ T_EM_ cells expressing a cytotoxic effector phenotype indicative for local antigenic stimulation. Second, T cells cultured from WML in four of nine MS patients recognize autoBLCL. This reactivity was profound in TCL generated from AL and mAIL. Third, no substantial T-cell reactivity was observed towards seven human cMSAg in CSF- and brain tissue-derived TCL of MS patients expressing the major MS-associated HLA risk alleles HLA-A*03, -DRB1*15 and -DRB1*13.

During the past decade, the focus on the role of CD4^+^ T cells in MS pathology has shifted towards CD8^+^ T cells; the most abundant T-cell subset identified in active WML of MS patients [12, 19]. The antigen specificity (e.g., autoantigens and/or EBV proteins) and the potential role of intra-lesional CD8^+^ T cells (cytotoxic or regulatory) in MS pathology is still a matter of debate [3, 22, 41, 54]. The infrastructure of the Netherlands Brain Bank and the commitment of MS patients to donate their tissues after death for research purposes, offered us the unique opportunity to compare the phenotype, function and reactivity of T cells between paired PB, CSF, NAWM and WML of 27 MS patients. Notably, the study design enabled comparison of T cells between compartments intra-individually, hereby limiting influence of inter-patient immune differences that are potentially unrelated to MS. A limitation of our study was, however, that all MS patients included had a long progressive disease course. Nevertheless, in situ characterization of the macroscopically defined NAWM and WML tissues obtained clearly demonstrated the presence of lesions covering the complete spectrum of MS disease activity ranging from DWMA to IL [29]. Markedly, about half of the macroscopically defined NAWM contained DWMA that represent periplaque abnormalities, Wallerian degeneration or pre-lesional abnormalities [29]. The relative high frequency of these type of diffuse abnormalities outside of focal MS lesions in end-stage MS patients raises doubt if all DWMA will progress to active lesions, which necessitates identification of specific markers that identifies the origin and fate of DWMA in time.

Concordant with previous studies, T cells were located in perivascular spaces of both NAWM and WML, whereas parenchymal T cells were restricted to WML of MS patients [41, 47]. The ex vivo distribution of CD8^+^ T_NA_, T_CM_, T_EM_ and T_EMRA_ cells in NAWM, WML and CSF of MS patients resembled data on WM and CSF under normal CNS conditions [16, 56]. However, compared to paired PB, CD8^+^ T cells in WML of the MS patients analyzed showed significantly increased expression of markers indicative for antigen-induced cytotoxicity (CD59L and grB) and activation (CD69, grB and CD137). The increased expression of the co-inhibitory (TIM3 and PD1) and co-stimulatory receptors (ICOS), whilst not accompanied with increased CD57 expression, suggests that the CD8^+^ T-cell underwent chronic activation in situ while retaining their proliferative potential. In comparison, CSF-derived CD8^+^ T cells had increased expression of both co-inhibitory markers, which refutes active involvement in CNS pathology [47]. Contrary to normal CNS conditions [56], CD8^+^ T cells in MS lesions expressed grB, suggesting that they encountered their cognate antigen locally and are potentially cytotoxic T cells [30, 38]. Indeed, occasionally CD8^+^ T cells with polarized grB expression were localized adjacent to cells showing signs of early apoptosis. However, detailed in situ analysis could not demonstrate a specific brain-resident cell type preferentially targeted by CD8^+^ T cells in the WML analyzed. Furthermore, the majority of WML-derived CD8^+^ T cells expressed CD69, which in the absence of CD103 co-expression may reflect their activated state or indicate a specific subset of T_RM_ cells [10, 27]. Overall, the activated and cytotoxic effector memory phenotype of CD8^+^ T cells in WML, along with overlapping TCRVβ repertoires between paired WML-TCL of the same patient, implicate clonally expanded T cells recognizing their cognate antigen locally. These CD8^+^ T cells are potentially involvement in the deleterious CNS inflammation that contributes to MS pathogenesis [38].

To decipher the cognate antigen driving T-cell activation in MS lesions, we analyzed T-cell reactivity in short-term TCL generated from different compartments of the same patient using autologous or alternatively an allogeneic HLA-matched BLCL stably transduced with seven human cMSAg (i.e., CNTN2, KIR4.1, MAG, MBP1, MOG, NFASC and S100B) [42, 43]. The expression of three well-described MS-associated HLA-I and -II risk alleles (i.e., HLA-A*0301, -DRB1*1501 and -DRB*1301; [50]) by the allogeneic BLCL-GR facilitated simultaneous detection of CD4^+^ and CD8^+^ T-cell responses in TCL of HLA-matched MS patients towards endogenously synthesized and processed human cMSAg [42]. Notably, no substantial cMSAg-specific T-cell reactivity was detected in TCL generated from CSF, NAWM and WML of 14 MS patients. The data concur with recent studies on CSF-TCL of patients with early MS describing negligible intrathecal cMSAg-specific T-cell reactivity [42, 62], but are in contrast to other studies showing brisk systemic and occasional local T-cell reactivity towards distinct cMSAg-specific synthetic peptides in MS patients [9, 14, 17, 18, 37]. These discrepancies may be methodological or related to the timing of tissue sampled during the course of disease. Most studies assayed cMSAg-specific T-cell reactivity using autologous PBMC as APC pulsed with high concentrations synthetic peptides (10–250 µM) or recombinant (animal) cMSAg. However, disease-irrelevant T cells expressing low-avidity TCR are potentially activated by these super-physiological peptide concentrations [7]. Also, due to species differences, amino acid composition and conformation of the synthetic peptides used may differ from natural T-cell epitopes [15]. The BLCL/cMSAg platform used in this study offered a more physiological approach to detect cMSAg-specific CD4^+^ and CD8^+^ T-cell responses as compared to more conventional APC platforms used previously [42]. Our inability to detect cMSAg-specific T-cell reactivity may be due to the inclusion of specimens from patients with advanced MS disease in which the disease-initiating cMSAg-specific T-cell response that potentially sparked of disease is not actively involved in perpetuating MS pathology. Alternatively, cMSAg-derived peptides were presented by other HLA alleles not shared with the allogenic BLCL-GR. Nevertheless, the inability to detect intra-lesional cMSAg-specific T-cell responses in end-stage MS patients, as well as in CSF of patients with clinical isolated syndrome and early MS [42], does not support the long-held hypothesis that the cMSAg assayed here are prominent targets of local pathogenic T-cell responses in MS patients.

Contrastingly, brisk T-cell reactivity (mainly CD8^+^ T cells) was detected towards autoBLCL, preferentially among TCL generated from AL and mAIL, but not DWMA and IL, suggesting their involvement in lesion activity. On occasion, these strong CD8^+^ T-cell responses were identified in TCL generated from anatomically separated WML of the same MS patient, suggesting involvement of the same TCC in both lesions. Indeed, even though observed in a single MS patient and not confirmed at the TCR level, the autoBLCL-reactive CD8^+^ T-cell response in the paired mAIL-TCL of this particular MS patient was dominated by TCRVβ2^+^ CD8^+^ T cells. These T cells were detected in the parenchyma of the respective WML tissues of the same patient with polarized TCR and CD8 expression suggesting immunological synapse formation in situ. Recently, we and others have shown intrathecal CD8^+^ T-cell reactivity to EBV antigens in patients with early MS [24, 31, 43]. Because no EBV transcripts could be detected in the corresponding WML assayed here, the autoBLCL-specific CD8^+^ T-cell responses identified are most likely not involved to combat intracerebral EBV infection as suggested by others [2, 53]. The cognate antigen could be an EBV protein, an autoantigen induced or selectively processed in EBV-infected B cells or an EBV/cMSAg-cross-reactive autoantigen [21, 23, 32, 36, 52, 61]. A limitation of our APC platform, using BLCL as APC, is that it withholds clear distinction between these options. Unfortunately, autologous activated B cells were not available to determine if the T-cell reactivity towards autoBLCL truly targeted EBV antigens. Furthermore, due to limited numbers of T cells available from the autoBLCL-reactive TCL, in-depth cognate EBV antigen discovery as described previously was also not possible [43].

In conclusion, the data presented suggest that cytotoxic CD8^+^ T_EM_ cells directed to autoBLCL, but not to the seven human cMSAg assayed in combination with three major MS-associated HLA risk alleles, are potentially involved in the immunopathology of white matter lesions of the MS patients analyzed. Follow-up studies on WML-derived T cells are warranted to identify the cognate virus and/or host antigen recognized by this potential deleterious autoBLCL-reactive T-cell response in MS patients.

## Electronic supplementary material

Below is the link to the electronic supplementary material.

**Online Resource 1** (PDF 105 kb)

**Online Resource 2** (PDF 89 kb)

**Online Resource 3** (PDF 2179 kb)

**Online Resource 4** (PDF 970 kb)

**Online Resource 5** (PDF 1229 kb)

**Online Resource 6** (PDF 588 kb)

**Online Resource 7** (PDF 1113 kb)

**Online Resource 8.** Video showing three-dimensional reconstruction of optically sectioned cryostat sections of surplus white matter lesion tissue (white matter lesion #2) of MS patient #6 (see Online Resource 1) stained with fluorochrome-conjugated antibodies to TCRVβ2 chain (green color), laminin (orange color) and CD8 (red color). Nuclei were stained with DAPI (blue color). TCRVβ2^+^ CD8^+^ T cells are located in both the perivascular cuff and brain parenchyma. Notably, the majority of parenchymal T cells show polarization of both CD8 and TCRVβ2. Image stack size is 75 (x)×75 (y)×8 (z) µm. Movie is obtained from same tissues stained as shown in Fig. 7. (MP4 2623 kb)
