# Supplementary material for: Phenotypic and functional characterization of T cells in white matter lesions of multiple sclerosis patients
Source: Acta Neuropathol. 2017 Jun 17;134(3):383–401. doi: 10.1007/s00401-017-1744-4 (PMC5563341; doi:10.1007/s00401-017-1744-4)
Supplement: Supplementary file 1 — Online Resource 1 (PDF 105 kb) [file 401_2017_1744_MOESM1_ESM.pdf]

Online Resource 1. General characteristics of multiple sclerosis patients and white matter brain tissues obtained for *ex vivo* and *in vitro* T-cell analyses.

Patient ID	Clinical course[a]	Gender[b]	Age (yrs)	Disease duration (yrs)[c]	EDSS[d]	Cause of death	CSF (pH)	PMI (hrs)[e]	IHC of viable tissues[f]		HLA class I[g]			HLA class II[g]	
									NAWM	Lesion(s)	A	B	C	DRB1	DQB1
1	SPMS	F	64	31	≥8	Urosepsis	6.20	10:10	NAWM	mAIL	*02, *03	*07, *51	*02, *07	*11, *15	*03, *06
2	PPMS	F	88	25	6-7	Exhaustion by chronic colitis ulcerosis	6.85	7:55	NAWM	mAIL	*01, *02	*07, *38	*07, *12	*15, *15	*06, *06
3	SPMS	M	45	10	≥8	Pulmonary embolisms and cardiac arrest	6.22	7:45	n.a.	mAIL	*01, *01	*07, *08	*07, *07	*03, *15	*02, *06
4	SPMS	M	76	44	≥8	Cerebrovascular incident	6.42	7:35	NAWM	DWMA	*01, *26	*27, *37	*01, *06	*10, *16	*05, *05
5	RRMS	F	59	24	7-8	Euthanasia	7.08	4:45	DWMA	AL	*11, *24	*35, *52	*03, *12	*01, *13	*05, *06
6	SPMS	F	68	37	≥8	Euthanasia	6.40	10:20	mAIL	mAIL	*02, *68	*14, *57	*06, *08	*13, *15	*03, *06
7	PPMS	M	83	21	n.d.	Pneumonia and lung cancer	6.40	7:50	DWMA	mAIL	*02, *29	*40, *45	*03, *06	*12, *13	*03, *06
8	SPMS	F	74	25	≥8	Cardio-respiratory insufficiency	6:43	10:15	NAWM	IL	*03, *03	*07, *44	*07, *16	*07, *15	*02, *06
9	PPMS	M	54	12	7-8	Euthanasia	6.39	8:15	n.a.	2xmAIL	*11, *29	*35, *44	*04, *16	*01, *15	*02, *06
10	PPMS	F	66	23	≥8	Euthanasia	6.45	9:35	NAWM	mAIL	n.d.	n.d.	n.d.	n.d.	n.d.
11	SPMS	F	56	32	≥8	Pneumonia	6.16	8:25	n.a.	AL	n.d.	n.d.	n.d.	n.d.	n.d.
12	RRMS	M	56	13	7-8	Suicide	7.10	10:10	DWMA	DWMA	n.d.	n.d.	n.d.	n.d.	n.d.
13	SPMS	M	73	43	≥8	Urosepsis	6.40	8:45	NAWM	IL	n.d.	n.d.	n.d.	n.d.	n.d.
14	PPMS	F	54	31	≥8	Heart Failure	6.27	9:20	DWMA	IL	n.d.	n.d.	n.d.	n.d.	n.d.
15	SPMS	M	54	25	≥8	Euthanasia	6.26	10:50	DWMA	mAIL	n.d.	n.d.	n.d.	n.d.	n.d.
16	SPMS	F	95	54	≥8	Infection, cachexia and dehydration	6.40	10:00	NAWM	AL	n.d.	n.d.	n.d.	n.d.	n.d.
17	n.d.	M	71	15	6.5	Metastasized pulmonary carcinoma	6.38	8:45	n.a.	n.d	*01, *02	*07, *07	*07, *07	n.d.	n.d.
18	SPMS	M	59	21	7	Euthanasia	6.50	10:45	DWMA	mAIL	n.d.	n.d.	n.d.	n.d.	n.d.
19	SPMS	F	53	16	7-8	Euthanasia	6.81	7:15	DWMA	DWMA	*02, *03	*07, *35	*04, *07	*01, *15	*05, *06
20	RRMS	F	87	18	≥8	Dehydration and renal insufficiency	5.92	9:30	NAWM	DWMA	*02, *02	*15, *44	*03, *07	*15, *15	*06, *06
21	SPMS	F	48	22	≥8	Respiratory failure	6.10	11:50	NAWM	mAIL	*01, *11	*15, *52	*03, *12	*04, *11	*03, *03
22	PPMS	M	66	25	≥8	Euthanasia	7.28	10:55	mAIL	AL	*02, *03	*07, *15	*03, *07	*15, *15	*06, *06
23	PPMS	M	57	25	6.5	Sepsis	6.80	10:15	NAWM	mAIL	n.d.	n.d.	n.d.	n.d.	n.d.
24	SPMS	F	56	21	6.5	Suicide	6.42	14:00	n.d.	n.d.	*24, *30	*18, *40	*03, *05	n.d.	n.d.
25	PPMS	F	51	15	≥8	Euthanasia	6.60	9:45	n.d.	n.d.	n.d.	n.d.	n.d.	n.d.	n.d.
26	n.d.	F	35	10	≥8	Euthanasia	6.37	10:20	n.d.	n.d.	n.d.	n.d.	n.d.	n.d.	n.d.
27	SPMS	F	74	50	>7.5	Euthanasia	6.40	7:50	NAWM	mAIL	n.d.	n.d.	n.d.	n.d.	n.d.

[a]RR, relapsing remitting MS; PP, primary progressive MS; SP, secondary progressive MS; Und., undefined disease course. [b]Gender: Male or Female, [c]Duration disease since clinical diagnosis of MS. [d]Expanded disability status scale. [e]Post-mortem interval: time between death and the end of autopsy. [f]Macroscopically characterized normal appearing white matter (NAWM) and MS lesion tissues obtained were subjected to immunohistochemistry (IHC) to classify WM as: NAWM, diffuse white matter abnormalities (DWMA), active lesion (AL), mixed active/inactive lesion (mAIL) and inactive lesion (IL) (see reference 29). n.a., not available; n.d., not determined. [g]HLA genotypes overlapping with BLCL-GR (HLA-A*01,*03, -B*07,*27, -C*02,*07, -DRB1*13,*15, -DQB1*06,*06) are underlined.
