# Supplementary material for: Phenotypic and functional characterization of T cells in white matter lesions of multiple sclerosis patients
Source: Acta Neuropathol. 2017 Jun 17;134(3):383–401. doi: 10.1007/s00401-017-1744-4 (PMC5563341; doi:10.1007/s00401-017-1744-4)
Supplement: Supplementary file 2 — Online Resource 2 (PDF 89 kb) [file 401_2017_1744_MOESM2_ESM.pdf]

Online Resource 2. General characteristics of multiple sclerosis patients and white matter brain tissues obtained for *in situ* T-cell analyses.

Patient ID[a]	Clinical course[b]	Gender[c]	Age (yrs)	Disease duration (yrs)[d]	EDSS[e]	Cause of death	CSF (pH)[f]	PMI (hrs)[g]	IHC of FFPE tissue(s)[h]
S99/128	PPMS	M	74	40	≥8	Resiratory insufficiency	6.91	8:30	IL
S00/274	SPMS	M	64	34	≥8	End stage MS	6.84	7:30	AL
S02/052	SPMS	F	75	34	7-8	Pneumonia	6.40	8:00	IL
S06/054	PPMS	F	44	6	≥8	Decompensation	6.34	10:15	IL
S06/139	SPMS	M	56	21	≥8	Pneumonia	6.65	8:00	mAIL
S07/216	SPMS	M	71	26	≥8	Pneumonia	6.60	7:00	mAIL
S09/251	SPMS	M	75	39	6.5	Euthanasia	6.53	7:45	mAIL
S10/020 (#6)	SPMS	F	68	37	≥8	Euthanasia	6.40	10:20	DWMA, AL & mAIL
S11/080 (#11)	SPMS	F	56	32	≥8	Pneumonia	6.16	8:25	mAIL
S12/008	SPMS	F	66	17	≥8	Pulmonary hypertension	6.73	10:45	AL

[a]Patient number as assigned by the Netherlands Brain Bank (#6 and #11 refer to patient ID in Online Resource 1).

[b]RR, relapsing remitting MS; PP, primary progressive MS; SP, secondary progressive MS; Und., undefined disease course.

[c]Gender: Male or Female.

[d]Duration disease since clinical diagnosis of MS.

[e]Expanded disability status scale.

[f]CSF, cerebrospinal fluid.

[g]Post-mortem interval: time between death and the end of autopsy.

[h]Formallin-fixed and paraffin-embedded (FFPE) white matter lesion tissues were characterized by immunohistochemistry (IHC) to classify the sampled WML as: diffuse white matter abnormality (DWMA), active lesion (AL), mixed active/inactive lesion (IAL) and inactive lesion (IL) (see reference 29).
