# Supplementary material for: Phenotypic and functional characterization of T cells in white matter lesions of multiple sclerosis patients
Source: Acta Neuropathol. 2017 Jun 17;134(3):383–401. doi: 10.1007/s00401-017-1744-4 (PMC5563341; doi:10.1007/s00401-017-1744-4)
Supplement: Supplementary file 3 — Online Resource 3 (PDF 2179 kb) [file 401_2017_1744_MOESM3_ESM.pdf]

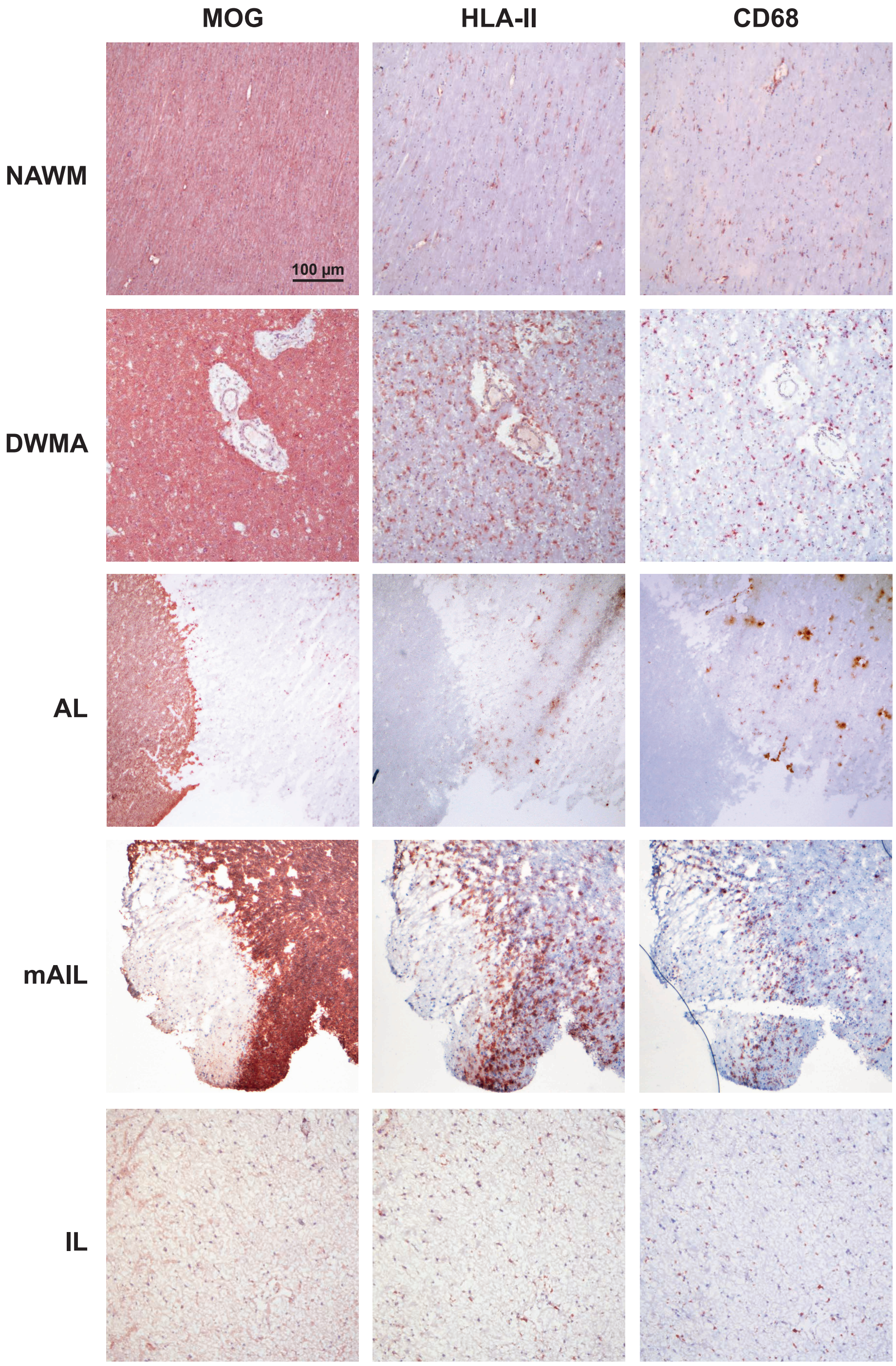

Online Resource 3. Examples of distinct white matter phenotypes of MS patients. Representative examples of MS white matter stainings according to Kuhlmann et al., Acta Neuropathol (2017) [29]. We used myelin oligodendrocyte glycoprotein (MOG), HLA class II (HLA-II) and CD68, expressed by macrophages and microglia, as markers to discriminate normal appearing white matter (NAWM: HLA-II$^{+/-}$Myelin^{+}CD68$^{+/-}$), diffuse white matter abnormalities (DWMA: HLA-II^{++}Myelin^{+}CD68^{+}), active lesions (AL: HLA-II^{++}Myelin$^{+/-}$CD68^{+}), mixed active/inactive lesions (mAIL; hypercellular rim: HLA-II^{++}Myelin$^{+/-}$CD68^{++} and hypocellular center: HLA-II$^{+/-}$Myelin^{-}CD68$^{+/-}$) and inactive lesions (IL: hypocellular: HLA-II$^{+/-}$Myelin^{-}CD68$^{+/-}$) in consecutive 8 µm sections of snap-frozen MS brain tissues. Scale bar is indicated in top left image.
