# Supplementary material for: Phenotypic and functional characterization of T cells in white matter lesions of multiple sclerosis patients
Source: Acta Neuropathol. 2017 Jun 17;134(3):383–401. doi: 10.1007/s00401-017-1744-4 (PMC5563341; doi:10.1007/s00401-017-1744-4)
Supplement: Supplementary file 4 — Online Resource 4 (PDF 970 kb) [file 401_2017_1744_MOESM4_ESM.pdf]

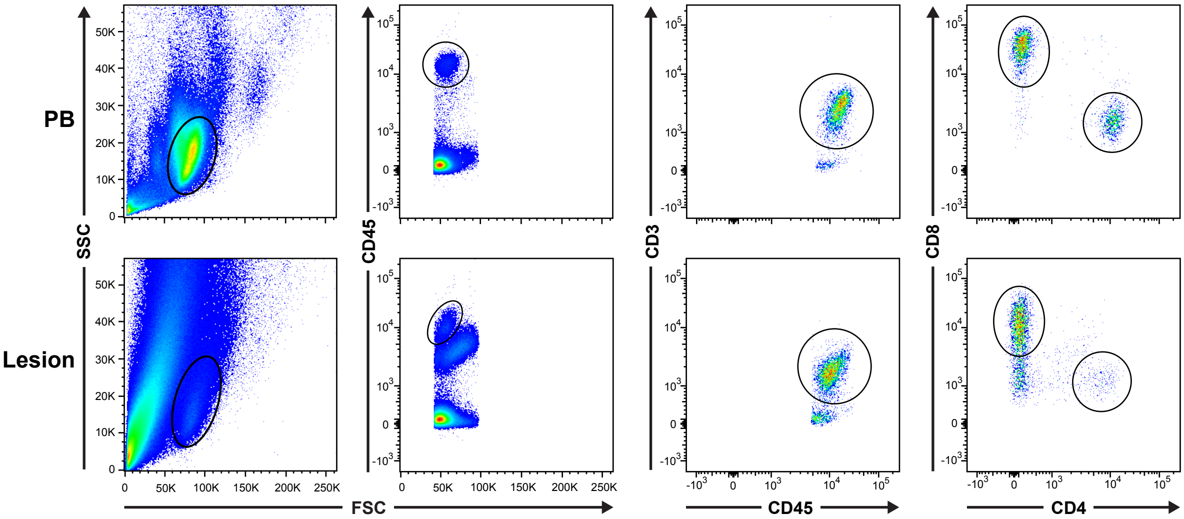

Online Resource 4. Gating procedure of paired PB- and white matter lesion-derived lymphocytes from MS patients. Lymphocytes were isolated from paired peripheral blood (PB), cerebrospinal fluid (CSF), normal-appearing white matter (NAWM) and white matter lesions (lesion) from patients with advanced MS (n=17) and subjected to multiplex flow cytometry. Pseudocolored density plots of paired PB (top panels) and white matter lesion (bottom panels) samples from one representative MS patient are shown. Lymphocytes were identified based on low forward, intermediate sideward scatter (FSC and SSC, respectively) and high CD45 expression, which was followed by subgating for CD3 and either high CD4 or high CD8 expression.
