# Supplementary material for: Phenotypic and functional characterization of T cells in white matter lesions of multiple sclerosis patients
Source: Acta Neuropathol. 2017 Jun 17;134(3):383–401. doi: 10.1007/s00401-017-1744-4 (PMC5563341; doi:10.1007/s00401-017-1744-4)
Supplement: Supplementary file 5 — Online Resource 5 (PDF 1229 kb) [file 401_2017_1744_MOESM5_ESM.pdf]

		Paired WML and NAWM								Paired WML and WML											
Patient		#2		#8		#21		#23		#5		#6		#7		#9		#19		#22	
CD4⁺ T-cells	PB healthy donors	mAIL	NAWM	IL	NAWM	mAIL	NAWM	mAIL	NAWM	AL	DWMA	mAIL1	mAIL2	AL	DWMA	mAIL1	mAIL2	DWMA 1	DWMA 2	AL	mAIL
TCRVß1	3.3	0.1	0.0	0.1	0.2	0.0	0.0	0.1	1.6	3.5	5.0	6.6	5.2	0.7	0.6	3.0	6.5	0.6	0.9	0.0	1.5
TCRVß2	9.4	1.3	0.1	14.4	2.8	45.3	0.2	0.2	13.0	6.2	3.3	5.9	0.0	15.8	3.6	24.4	16.1	14.4	9.0	1.2	4.1
TCRVß3	4.4	0.1	12.5	0.1	9.8	0.0	0.0	3.1	0.4	0.2	0.0	0.0	3.1	3.8	3.6	2.6	2.6	2.4	1.1	0.6	1.7
TCRVß4	2.0	0.0	52.0	0.0	0.0	0.0	0.0	0.0	0.3	2.0	0.0	0.0	0.0	0.0	0.0	0.1	0.0	6.7	0.0	0.0	0.0
TCRVß5.1	6.7	0.0	0.1	3.4	0.5	0.0	23.8	0.0	0.4	1.3	2.8	0.0	0.0	5.4	1.5	0.1	0.1	0.0	1.7	0.4	11.9
TCRVß5.2	1.3	0.0	0.0	0.5	1.5	0.0	0.0	0.0	0.0	1.5	0.5	0.6	0.0	0.7	0.3	0.3	1.8	0.1	1.7	0.4	0.2
TCRVß5.3	1.1	0.1	0.0	0.2	0.5	0.2	0.0	0.0	0.0	0.7	0.0	0.2	0.0	0.2	3.6	1.8	0.6	0.0	0.2	0.3	0.0
TCRVß7.1	1.9	0.0	0.0	3.7	2.1	0.0	0.0	23.0	0.0	1.3	0.0	0.0	0.0	0.6	0.8	0.7	0.2	2.1	4.7	0.3	0.1
TCRVß7.2	1.1	0.0	0.0	0.3	0.0	0.0	0.0	0.1	0.0	0.8	0.0	2.0	0.1	0.0	0.2	0.1	0.0	0.0	2.2	0.6	4.3
TCRVß8	4.8	1.6	0.0	6.0	0.1	25.7	0.0	0.4	1.3	1.0	11.8	0.2	0.1	1.9	1.0	3.8	4.7	4.3	0.9	1.0	1.0
TCRVß9	4.1	0.0	0.0	11.5	0.0	17.1	0.5	6.8	0.0	4.6	0.0	0.4	0.2	0.6	6.9	2.1	2.7	0.8	0.0	0.4	0.0
TCRVß11	0.9	0.3	0.8	0.2	0.2	0.3	0.8	0.5	0.3	0.4	0.1	0.3	0.2	0.3	0.1	0.3	0.1	0.3	5.4	1.2	0.2
TCRVß12	1.8	0.1	9.6	0.2	1.3	0.1	0.0	0.1	0.0	1.6	0.0	0.0	0.0	1.3	2.4	0.4	1.9	0.3	0.2	0.0	0.3
TCRVß13.1	4.0	0.0	1.0	33.4	0.3	0.9	2.1	0.8	21.3	11.9	0.4	0.6	0.1	1.6	1.2	0.7	1.7	0.6	1.0	2.1	3.4
TCRVß13.2	2.8	0.0	0.2	0.2	2.8	0.0	0.1	0.0	15.5	1.6	10.8	0.4	0.1	0.3	0.6	0.4	0.4	2.5	0.2	0.0	1.4
TCRVß13.6	1.9	82.7	9.8	0.5	0.4	0.0	0.0	0.0	4.1	10.3	5.6	2.2	0.0	0.9	1.0	3.4	0.1	4.4	1.1	0.0	1.0
TCRVß14	2.6	2.3	0.1	0.1	0.0	0.0	6.9	2.0	0.0	0.2	3.1	7.3	0.0	2.6	5.5	1.8	1.9	0.2	0.0	0.6	1.1
TCRVß16	1.0	0.0	0.0	0.0	1.1	0.0	0.0	1.3	0.0	3.7	1.3	0.3	0.1	0.2	8.0	0.7	0.0	0.5	0.0	0.4	0.0
TCRVß17	5.5	0.1	0.1	1.7	3.1	0.0	0.0	0.9	1.7	1.3	0.7	5.7	0.0	3.7	2.0	5.2	10.2	1.8	2.6	0.0	4.9
TCRVß18	1.9	0.1	0.1	0.2	0.0	3.8	0.0	0.0	0.7	0.2	0.2	0.2	0.0	0.2	0.3	0.1	0.2	0.0	0.2	0.0	0.2
TCRVß20	2.6	0.1	4.9	0.3	0.1	1.7	54.6	30.3	0.0	2.7	10.4	9.5	0.0	0.2	1.4	7.7	19.0	1.9	11.6	82.9	0.4
TCRVß21.3	2.5	0.0	0.0	0.0	0.1	0.0	0.0	0.0	0.6	0.7	0.4	0.1	0.8	4.7	2.3	0.7	1.3	2.3	5.8	0.0	0.0
TCRVß22	4.3	0.0	0.0	0.1	0.1	0.0	0.7	0.0	3.0	1.0	1.9	0.6	62.2	1.6	5.1	1.8	3.7	0.3	1.6	0.0	8.1
TCRVß23	0.5	0.0	0.0	0.0	0.0	0.0	0.0	0.2	0.0	0.1	0.3	0.0	0.0	0.0	0.0	0.1	0.0	0.0	0.0	0.3	0.0
undetermined	27.8	11.0	8.9	22.9	73.0	4.8	10.1	30.2	35.9	41.4	41.5	57.0	27.6	52.8	48.2	37.8	24.4	53.4	48.0	7.3	54.3
														r = 0.50<br>p = 0.01		r = 0.79<br>p = 0.0001					

CD8⁺ T-cells	PB healthy donors	#2 mAIL	#2 NAWM	#8 IL	#8 NAWM	#21 mAIL	#21 NAWM	#23 mAIL	#23 NAWM	#5 AL	#5 DWMA	#6 mAIL1	#6 mAIL2	#7 AL	#7 DWMA	#9 mAIL1	#9 mAIL2	#19 DWMA 1	#19 DWMA 2	#22 AL	#22 mAIL
TCRVß1	4.2	0.0	0.1	3.6	0.1	36.3	0.1	0.0	0.1	14.9	1.0	0.0	0.0	1.7	0.3	2.2	1.4	28.2	0.2	0.0	4.3
TCRVß2	5.4	8.1	1.9	2.2	0.4	23.3	3.8	0.0	0.0	5.2	0.6	23.1	58.0	7.3	16.8	5.0	5.7	7.4	0.1	0.1	1.4
TCRVß3	4.4	0.1	3.7	0.0	4.2	0.0	0.0	0.1	0.1	0.0	0.0	9.7	0.2	19.6	3.1	8.0	2.3	0.2	4.5	0.0	0.0
TCRVß4	1.9	0.0	0.0	0.0	0.1	0.0	0.0	0.0	0.1	0.0	0.0	0.0	0.0	0.0	0.0	0.0	0.0	0.0	0.0	0.0	0.0
TCRVß5.1	3.2	0.0	0.0	0.4	11.2	1.4	0.0	0.0	0.0	1.8	0.5	0.0	0.0	1.2	3.7	0.2	0.0	0.2	0.1	0.0	4.4
TCRVß5.2	1.1	0.2	0.2	0.2	0.1	0.0	0.0	0.0	0.0	0.7	0.0	0.0	0.0	1.2	0.0	0.2	0.0	0.0	0.0	0.0	0.2
TCRVß5.3	0.9	0.1	0.7	0.7	0.2	4.0	0.0	0.0	0.0	0.0	0.0	0.0	0.0	0.0	0.0	0.3	2.0	0.0	3.5	0.0	0.7
TCRVß7.1	3.4	1.1	0.0	0.1	0.0	0.0	0.0	0.0	0.0	0.1	0.0	0.0	0.0	3.2	16.5	0.3	0.7	0.1	0.1	0.0	0.0
TCRVß7.2	2.4	0.1	0.0	0.0	0.0	0.0	0.0	0.0	0.2	1.4	0.2	0.0	0.0	0.1	0.2	0.2	0.0	0.0	0.5	0.0	0.0
TCRVß8	4.1	0.0	0.0	3.2	0.0	1.3	30.4	0.0	0.1	0.2	0.0	23.2	10.0	7.6	1.2	6.8	5.7	1.1	0.1	0.0	0.0
TCRVß9	3.5	0.0	0.1	0.0	0.1	0.0	0.0	0.0	0.0	1.6	0.0	2.9	0.1	3.8	0.0	0.4	0.9	0.1	0.0	0.0	6.5
TCRVß11	0.9	0.2	0.0	0.2	0.0	0.0	14.8	0.2	0.1	0.2	0.0	0.4	0.3	0.8	0.1	0.3	0.0	0.2	0.5	0.2	0.0
TCRVß12	1.3	0.0	0.0	0.0	0.1	0.0	0.0	0.1	0.1	0.6	0.0	0.0	0.0	3.4	0.6	2.9	2.9	0.0	0.0	0.0	0.0
TCRVß13.1	3.4	0.1	0.0	0.0	0.0	0.0	0.1	0.1	0.1	0.9	7.2	4.9	0.0	4.3	1.3	1.4	2.3	0.1	0.5	40.4	1.0
TCRVß13.2	3.3	0.0	0.1	0.0	0.1	0.0	4.4	0.4	0.2	0.2	0.0	0.0	0.1	0.1	0.3	1.0	0.7	0.0	16.1	0.0	2.2
TCRVß13.6	1.6	0.3	16.9	0.3	11.8	0.4	0.1	0.0	0.0	2.2	0.1	0.2	0.0	6.4	0.2	0.0	0.2	1.1	0.4	0.0	1.8
TCRVß14	5.7	25.1	65.6	2.3	0.5	27.4	0.0	46.7	1.0	9.0	45.1	1.8	0.1	0.5	15.9	1.5	11.6	23.1	0.4	27.8	7.5
TCRVß16	0.8	0.0	0.1	0.0	0.4	1.7	0.0	0.0	0.2	0.0	0.0	0.0	0.1	0.5	4.1	0.1	0.6	14.4	0.3	13.3	0.0
TCRVß17	5.1	0.2	0.0	0.4	0.1	0.0	3.5	0.0	0.0	7.1	0.3	0.1	0.0	1.4	2.3	5.6	0.1	0.3	29.0	0.0	47.9
TCRVß18	0.6	0.1	0.0	0.1	0.0	0.0	0.0	0.0	0.0	0.0	0.0	0.0	0.0	0.1	0.0	0.0	0.0	0.0	0.1	0.0	0.0
TCRVß20	2.3	0.1	0.0	0.0	0.0	0.0	0.0	0.0	0.1	0.8	0.5	0.3	0.0	0.0	0.0	3.1	3.3	0.0	0.0	0.0	0.4
TCRVß21.3	2.4	0.0	0.0	1.5	0.2	0.5	3.0	0.0	0.1	1.9	0.0	1.4	0.7	1.0	0.4	1.4	0.7	18.0	2.8	0.0	0.3
TCRVß22	3.2	0.3	0.2	0.0	72.7	0.0	4.1	0.1	0.1	7.7	0.3	0.0	0.0	0.3	1.9	0.7	1.3	0.5	0.0	0.1	0.0
TCRVß23	1.3	0.0	3.0	0.3	0.8	0.0	0.0	0.0	0.0	0.3	0.0	0.4	0.0	0.6	0.2	2.8	2.2	0.0	0.0	0.0	0.0
undetermined	33.4	63.8	7.6	84.3	0.0	3.8	35.6	52.3	97.4	43.2	44.0	31.5	30.2	35.1	30.8	55.7	55.1	4.9	40.6	17.8	21.4
										r = 0.76<br>p = 0.0001		r = 0.65<br>p = 0.0007		r = 0.45<br>p = 0.03		r = 0.47<br>p = 0.02					

Online Resource 5. Correlation in T-cell receptor variable beta chain protein expression of CD4⁺ and CD8⁺ T-cells expanded from anatomically distinct white matter lesions of the same MS patient. Short-term T-cell lines (TCL) were generated by non-specific stimulation of T-cells recovered from paired white matter brain tissues that were histologically classified as normal-appearing white matter (NAWM) and white matter lesios (WML) classes; diffuse white matter abnormalities (DWMA), active lesions (AL), mixed active/inactive lesions (mAIL) and inactive lesions (IL) (see Online Resource 2 for criteria applied for MS WM classification). The T-cell receptor variable beta chain (TCRVβ) protein expression profile was determined for 10 MS patients by flow cytometry using monoclonal antibodies (mAb) directed to 24 distinct human TCRVβ chains for CD4⁺ (upper rows) and CD8⁺ T-cells (lower rows) of the indicated TCL for each MS patient (column pairs). Light to dark color grading indicate the lowest to highest frequencies of specific TCRVβ expressed by CD4⁺ and CD8⁺ T-cells in each TCL. The mean TCRVβ chain expression of PB-derived CD4⁺ and CD8⁺ T-cells from 85 unrelated healthy individuals are provided as reference (data provided by Beckman Coulter). "Undetermined" refers to T-cells expressing a TCRVβ chain not covered by the TCRVβ-specific monoclonal antibody panel used. Spearman's rank correlation test were used for statistical analysis.
