# Supplementary material for: Phenotypic and functional characterization of T cells in white matter lesions of multiple sclerosis patients
Source: Acta Neuropathol. 2017 Jun 17;134(3):383–401. doi: 10.1007/s00401-017-1744-4 (PMC5563341; doi:10.1007/s00401-017-1744-4)
Supplement: Supplementary file 6 — Online Resource 6 (PDF 588 kb) [file 401_2017_1744_MOESM6_ESM.pdf]

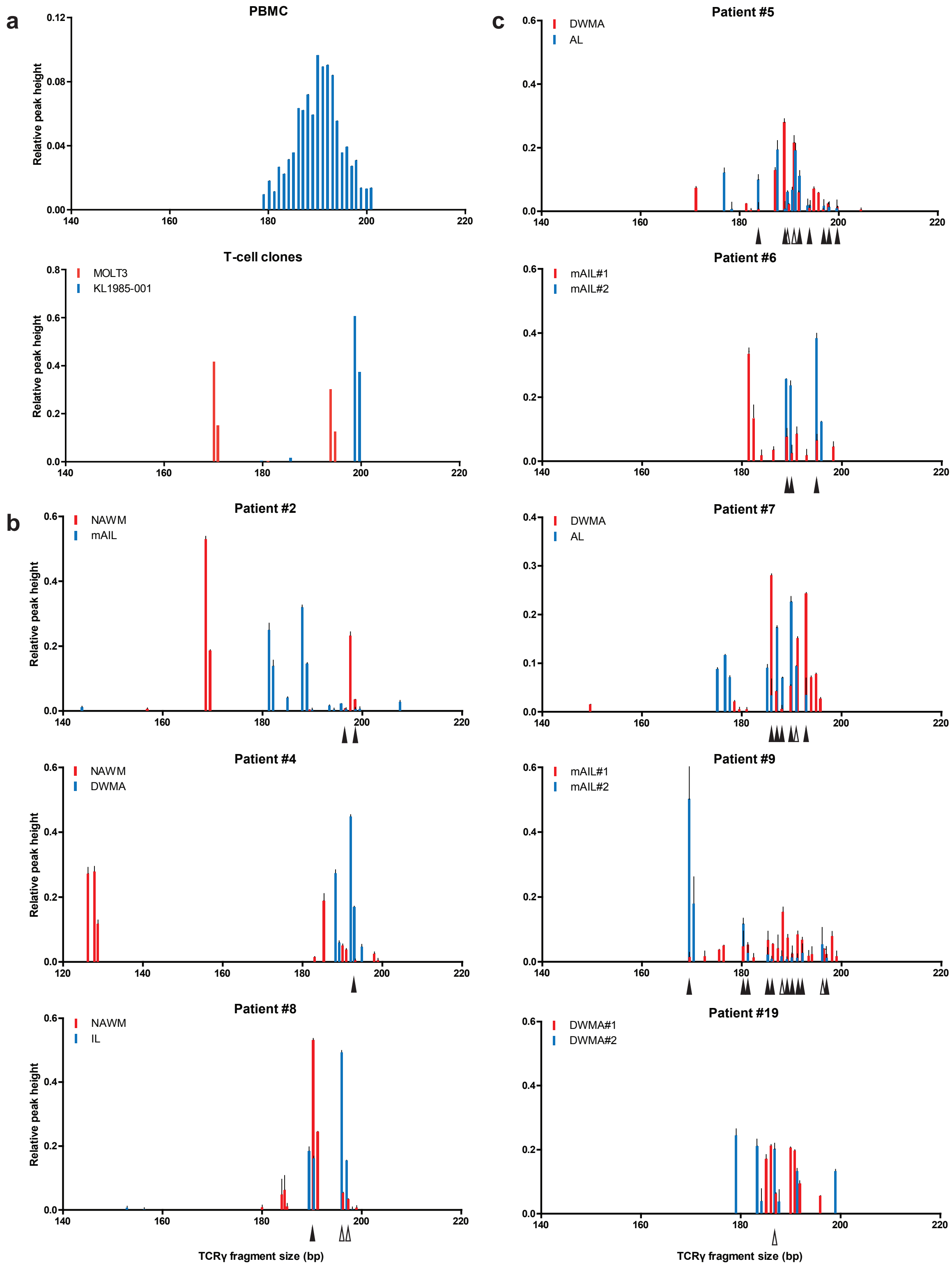

Online Resource 6. Increased clonal overlap in paired white matter lesions compaired to normal appearing white matter derived CD8⁺ T-cells in MS patients. The T-cell receptor gamma chain (TCRγ) rearrangement spectra was determined by multiplex PCR and fragment length analysis on DNA isolated from (**a**) peripheral blood mononuclear cells (PBMC, top panel) and two T-cell clones (bottom panel) with one (blue bars; KL1985-001) or two rearranged alleles (red bars; MOLT-3). Both TCC display additional +1 base pair (bp) peaks due to the addition of a 3'-adenosine residue by the non-proof reading polymerase used. TCRγ rearrangement spectra of DNA isolated from 10.000-200.000 sorted viable CD8⁺ T-cells of short-term T-cell lines generated from (**b**) immunohistologically classified paired normal appearing white matter (NAWM; red bars), diffuse white matter abnormalities (DWMA), active lesions (AL), mixed active/inactive lesions (mAIL) and inactive lesions (IL) (all white matter lesions shown as blue bars) of 3 MS patients and (**c**) paired white matter lesions (blue and red bars) of 5 MS patients are shown. The avarage size and peak height (bars) and peak height range (vertical line) of two independent PCRs are shown. Overlapping fragments are indicated by black (0-0.25 bp length difference) and white arrow heads (0.25-0.5 bp lenth difference).
