# Supplementary material for: Phenotypic and functional characterization of T cells in white matter lesions of multiple sclerosis patients
Source: Acta Neuropathol. 2017 Jun 17;134(3):383–401. doi: 10.1007/s00401-017-1744-4 (PMC5563341; doi:10.1007/s00401-017-1744-4)
Supplement: Supplementary file 7 — Online Resource 7 (PDF 1113 kb) [file 401_2017_1744_MOESM7_ESM.pdf]

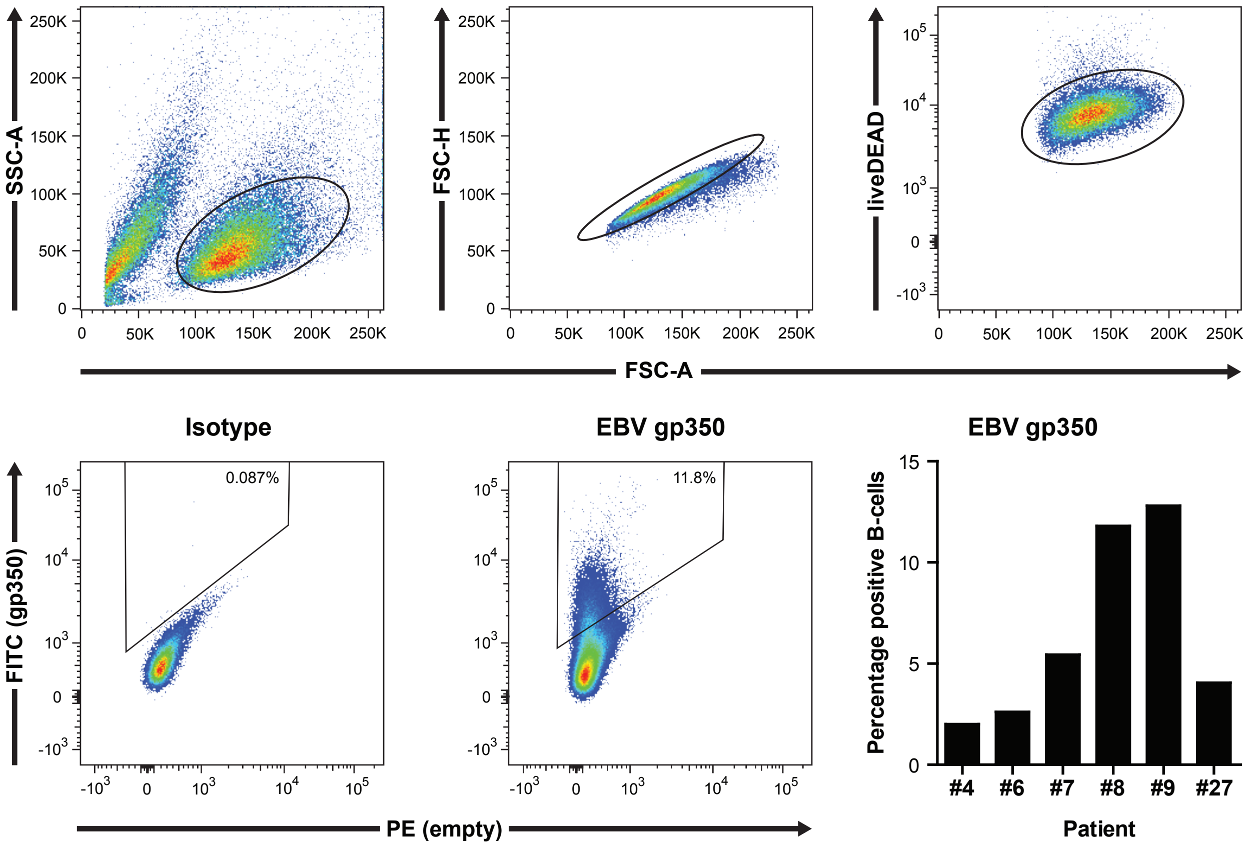

Online Resource 7. Low frequency of cultured latently Epstein-Barr virus infected human B-cell lines express the viral glycoprotein 350. Long-term cultured EBV-infected B-cell lines (BLCL) were stained using fluorescent viability marker and a monoclonal antibody (moAb) specific for EBV glycoprotein 350 (gp350), or as control an IgG1 isotype moAb, and staining visualized using a FITC-conjugated rabbit-anti- mouse IgG secondary antibody. EBV-specific glycoprotein 350 is expressed on the cell surface of EBV-infected B-cells undergoing productive virus infection, but not on latently EBV-infected B-cells. Single viable B-cells were gated based on sideward scatter (SSC-A) and forward scatter area (FSC-A) and height (FSC-H), intermediate liveDEAD staining. Gating of donor #8 is shown representative for 6 donors analysed.
